# Modest overexpression of *FOXO* maintains cardiac proteostasis and ameliorates age‐associated functional decline

**DOI:** 10.1111/acel.12543

**Published:** 2017-01-16

**Authors:** Anna C. Blice‐Baum, Alexander C. Zambon, Gaurav Kaushik, Meera C. Viswanathan, Adam J. Engler, Rolf Bodmer, Anthony Cammarato

**Affiliations:** ^1^ Division of CardiologyDepartment of MedicineJohns Hopkins UniversityBaltimoreMD21205USA; ^2^ Department of Biopharmaceutical SciencesKeck Graduate InstituteClaremontCA91711USA; ^3^ Sanford Burnham Prebys Medical Discovery Institute, Development, Aging and Regeneration ProgramLa JollaCA92037USA; ^4^ Department of BioengineeringUniversity of California, San DiegoLa JollaCA92093USA

**Keywords:** FOXO, protein homeostasis, *Drosophila*, cardiac aging, autophagy, UPS

## Abstract

Heart performance declines with age. Impaired protein quality control (PQC), due to reduced ubiquitin‐proteasome system (UPS) activity, autophagic function, and/or chaperone‐mediated protein refolding, contributes to cardiac deterioration. The transcription factor FOXO participates in regulating genes involved in PQC, senescence, and numerous other processes. Here, a comprehensive approach, involving molecular genetics, novel assays to probe insect cardiac physiology, and bioinformatics, was utilized to investigate the influence of heart‐restricted manipulation of *dFOXO* expression in the rapidly aging *Drosophila melanogaster* model. Modest *dFOXO* overexpression was cardioprotective, ameliorating nonpathological functional decline with age. This was accompanied by increased expression of genes associated predominantly with the UPS, relative to other PQC components, which was validated by a significant decrease in ubiquitinated proteins. RNAi knockdown of UPS candidates accordingly compromised myocardial physiology in young flies. Conversely, excessive *dFOXO* overexpression or suppression proved detrimental to heart function and/or organismal development. This study highlights *D. melanogaster* as a model of cardiac aging and FOXO as a tightly regulated mediator of proteostasis and heart performance over time.

## Introduction

The aging human heart exhibits functional decline and increased vulnerability to cardiovascular disease, the leading cause of death worldwide (Dai *et al*., 2012; Strait & Lakatta, 2012). Age‐related changes include decreased cardiac output, elevated susceptibility to arrhythmia, and impaired relaxation with increased myocardial stiffness. Reduced protein homeostasis, a highly conserved hallmark of aging, is associated with cellular dysfunction over time due to decreased function of protein quality control (PQC) processes including those mediated by the ubiquitin‐proteasome system (UPS), by the autophagy‐lysosomal pathway, and by chaperone‐mediated protein refolding (Lavandero *et al*., 2013; Lopez‐Otin *et al*., 2013; Willis & Patterson, 2013). Impaired proteostasis is particularly detrimental to cardiomyocytes since they exhibit limited regenerative capacity and thus rely heavily on PQC mechanisms for retarding functional decline throughout life (van Berlo & Molkentin, 2014; Wang & Robbins, 2014; Quarles *et al*., 2015).

The FOXO family of forkhead transcription factors, known modulators of aging and longevity, is involved in regulating genes associated with all three PQC processes (Tower, 2011; Paula‐Gomes *et al*., 2013; Webb & Brunet, 2014). The UPS is the first line of defense against cellular protein toxicity. Misfolded or damaged proteins are post translationally marked for degradation by ubiquitination (Powell, 2006; Wang & Robbins, 2014; Wang & Wang, 2015). Ubiquitinated substrates are then recognized and processed by the proteasome, a multi‐subunit complex that degrades proteins into amino acids, peptides, and ubiquitin. Inadequate UPS function has been increasingly identified as a contributor to proteotoxicity during aging and disease within all cell types including cardiac myocytes (Bulteau *et al*., 2002; Paula‐Gomes *et al*., 2013; Wang & Wang, 2015). Autophagy eliminates misfolded protein aggregates and dysfunctional organelles by lysosomal degradation (Lavandero *et al*., 2013; Lopez‐Otin *et al*., 2013; Wang & Robbins, 2014; Quarles *et al*., 2015) and has been implicated in the regulation of cardiomyocyte size and homeostasis during development and times of stress (Sengupta *et al*., 2011; Lavandero *et al*., 2013; Paula‐Gomes *et al*., 2013). Recent evidence suggests that the UPS and autophagy are highly dependent upon each other (Wang & Robbins, 2014; Wang & Wang, 2015). For example, under pathological conditions, both the UPS and autophagy tend to be activated simultaneously to manage proteolytic stress (Wang & Robbins, 2014; Wang & Wang, 2015). Chaperone‐mediated protein refolding also protects against cytotoxicity by preventing misfolded proteins from forming aggregates and has been linked to both senescence and cardiac disease (Calderwood *et al*., 2009; Willis & Patterson, 2010).

Since FOXOs are intimately connected with proteostasis, exploiting their therapeutic potential has previously been advocated (Maiese *et al*., 2009; Sengupta *et al*., 2011; Webb & Brunet, 2014). However, this potential may be limited due to the transcription factors' involvement in a complex myriad of biological processes, which have the capacity to serve both beneficial and detrimental roles under particular cellular contexts (Zhu *et al*., 2007; Rothermel & Hill, 2008; Ferdous *et al*., 2010; Ronnebaum & Patterson, 2010). For example, depending upon the level of FOXO‐mediated stimulation, autophagic activity in cardiomyocytes may be adaptive and protective or maladaptive as characterized by excessive catabolism, mitochondrial elimination, adverse remodeling, and reduced cell survival, all of which influence cardiac homeostasis (Zhu *et al*., 2007; Rothermel & Hill, 2008; Ferdous *et al*., 2010). Thus, FOXOs' therapeutic benefits, within myocardium specifically, may be dose‐dependent, which must be thoroughly vetted, ideally in a single animal model, to more completely comprehend their role in cardiovascular health.

*Drosophila melanogaster* is an ideal organism for studying nonpathological aging and the associated changes in heart function. Flies have short lifespans (~2 months), the capacity to produce large, isogenous populations of offspring, are compliant to extensive genetic manipulation, and recapitulate a number of hallmarks of cardiac senescence while simultaneously minimizing confounding environmental factors (Paternostro *et al*., 2001; Wessells *et al*., 2004; Cammarato *et al*., 2008; Nishimura *et al*., 2014; Kaushik *et al*., 2015). The simple dorsal vessel of the fruit fly contains ~100 cardiomyocytes, resembles the embryonic vertebrate heart, and consists of many of the same major cellular constituents including cytoskeletal, mitochondrial, metabolic, signaling, and PQC elements (Wessells *et al*., 2004; Cammarato *et al*., 2011; Nishimura *et al*., 2014). The throng of advanced molecular tools available in *D. melanogaster*, including the UAS‐GAL4 system (Brand & Perrimon, 1993), provides advantages for controlling dosage, timing, and tissue specificity of gene overexpression and knockdown.

Flies possess a single *FOXO* gene, *dFOXO*, which encodes multiple splice variants that give rise to distinct polypeptides. The longest dFOXO isoform shares 33% total protein sequence identity to human FOXO3 (89% identity to the forkhead box domain) and 31% total identity to human FOXO1 (88% identity to the forkhead box domain), the most abundant forkhead subtype O transcription factors expressed in the vertebrate heart (Ronnebaum & Patterson, 2010). Earlier studies revealed that *dFOXO*‐overexpression throughout all fly musculature affords increased lifespan, systemic preservation of proteostasis mediated by enhanced autophagy, and in the heart, defense against pacing‐induced failure at old age (Wessells *et al*., 2004; Demontis & Perrimon, 2010). However, these studies did not account for dFOXO dose‐responses, nor did they thoroughly scrutinize cardiac physiology or consider a protective role involving the UPS.

Although the benefits that FOXOs offer skeletal and cardiac myocytes have been shown in basal and disease states using a diverse compendium of cellular and animal models, their activity can also promote cellular injury and negatively impact viability (Zhu *et al*., 2007; Rothermel & Hill, 2008; Maiese *et al*., 2009; Demontis & Perrimon, 2010; Ferdous *et al*., 2010; Ronnebaum & Patterson, 2010; Sengupta *et al*., 2011; Webb & Brunet, 2014). To provide a more unified perspective, we aimed to comprehensively investigate the extent to which *FOXO* expression in myocardium provides defense against normal age‐associated decline and the influence of FOXO dosage to this effect in a single model organism. Here, we show that overexpression of *dFOXO* exclusively in *D. melanogaster* cardiomyocytes has dose‐dependent consequences on heart function and even organismal development. Moreover, we demonstrate that only in modest amounts does dFOXO provide protection against age‐related cardiac decline likely through enhanced UPS activity.

## Results

### Overexpression of *dFOXO* exclusively in the heart using GMH5‐GAL4 ameliorates age‐related functional decline

**Figure 1:**
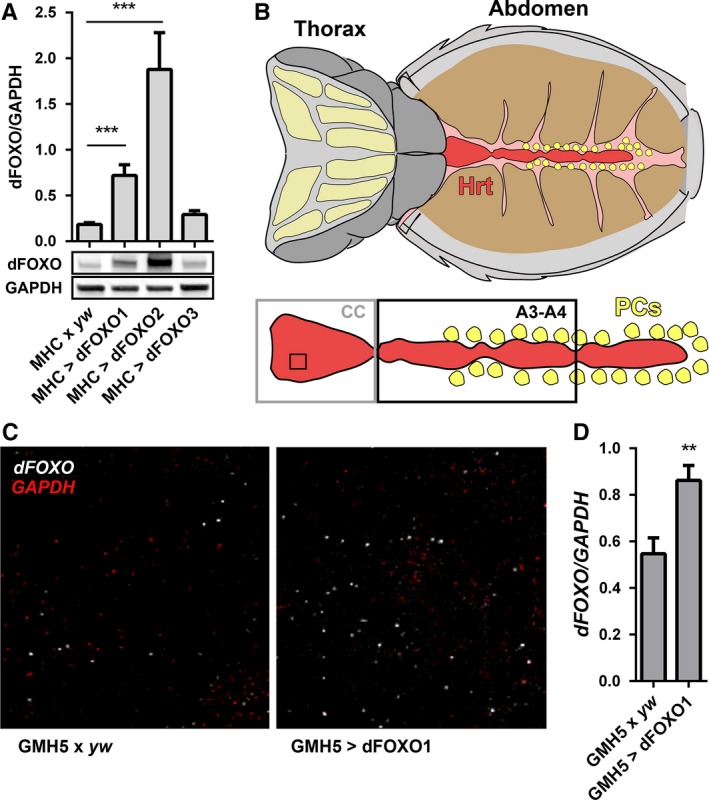
Overexpression of *dFOXO* in the *Drosophila melanogaster* heart. (A) *dFOXO* overexpression was assessed in skeletal muscle from three transgenic lines. Relative thoracic dFOXO content was determined via quantitative western blot analysis and compared to controls (MHC x *yw*) (*n* = 8, ***P <* *0.001, one‐way ANOVA, Bonferroni post‐hoc test). (B) Illustration of the semi‐intact *D. melanogaster* dorsal vessel, or heart tube, preparation with the heart (Hrt) shown in red, the conical chamber (CC) boxed in gray, abdominal segments three and four (A3‐A4) boxed in black, and the pericardial cells (PCs) depicted in yellow (modified from Vogler & Ocorr, 2009). (C) Fluorescence *in situ* hybridization was used to visualize and quantify GMH5‐GAL4‐mediated *dFOXO* overexpression exclusively in the cardiomyocytes. The confocal images shown represent an area from the CC denoted by the black square in 1B. White particles are individual *dFOXO* messages and red particles individual *GAPDH* messages. The latter served as an endogenous control. (D) *dFOXO* and *GAPDH* transcripts were quantified from confocal micrographs using the ImageJ particle counter (see materials and methods). *dFOXO*/*GAPDH* in *GMH5‐GAL4 > UAS‐dFOXO* line 1 hearts was significantly higher than in control (*GMH5‐GAL4 x yw*) hearts (*n* = 20 hearts, **P <* *0.01, Student's *t*‐test).

Three *UAS‐dFOXO D. melanogaster* lines were obtained that permit targeted transgene expression. To estimate the relative extent of overexpression afforded by the *UAS‐dFOXO* lines, each was independently crossed with *MHC‐GAL4*, a muscle‐specific driver line. The progeny expressed *UAS‐dFOXO* in tissues readily amenable to quantitative protein analysis. Thoracic musculature from *MHC‐GAL4 > UAS‐dFOXO* lines 1 and 2 contained approximately 4‐ (dFOXO/GAPDH = 0.72 ± 0.12 vs. 0.18 ± 0.02) and 10‐times (dFOXO/GAPDH = 1.88 ± 0.40 vs. 0.18 ± 0.02) the amount of endogenous dFOXO found in *MHC‐GAL4* x *yw* controls, respectively (Fig. 1A). A third transgenic line was found not to overexpress *dFOXO* significantly beyond endogenous levels (dFOXO/GAPDH = 0.29 ± 0.40 vs. 0.18 ± 0.02). Therefore, lines 1 and 2 were employed for subsequent *dFOXO* overexpression in the fly heart (Fig. 1B). To confirm cardiomyocyte‐specific *UAS‐dFOXO* overexpression by the GMH5‐GAL4 driver, individual *dFOXO* transcripts were visualized and quantified using high‐resolution fluorescence *in situ* hybridization (Figs 1C and S1). The resulting normalized *dFOXO* signal was ~55% higher in *GMH5‐GAL4 *>* UAS‐dFOXO* line 1 cardiomyocytes relative to that in *GMH5‐GAL4 x yw* controls (*dFOXO/GAPDH* = 0.86 ± 0.06 vs. 0.55 ± 0.07) (Fig. 1D). Attempts to resolve elevated nuclear dFOXO in *GMH5‐GAL4 *>* UAS‐dFOXO* line 1 myocardium via immunohistochemistry, however, yielded inconclusive results potentially due to low protein overexpression levels and/or reagent limitations (Fig. S2).

**Figure 2:**
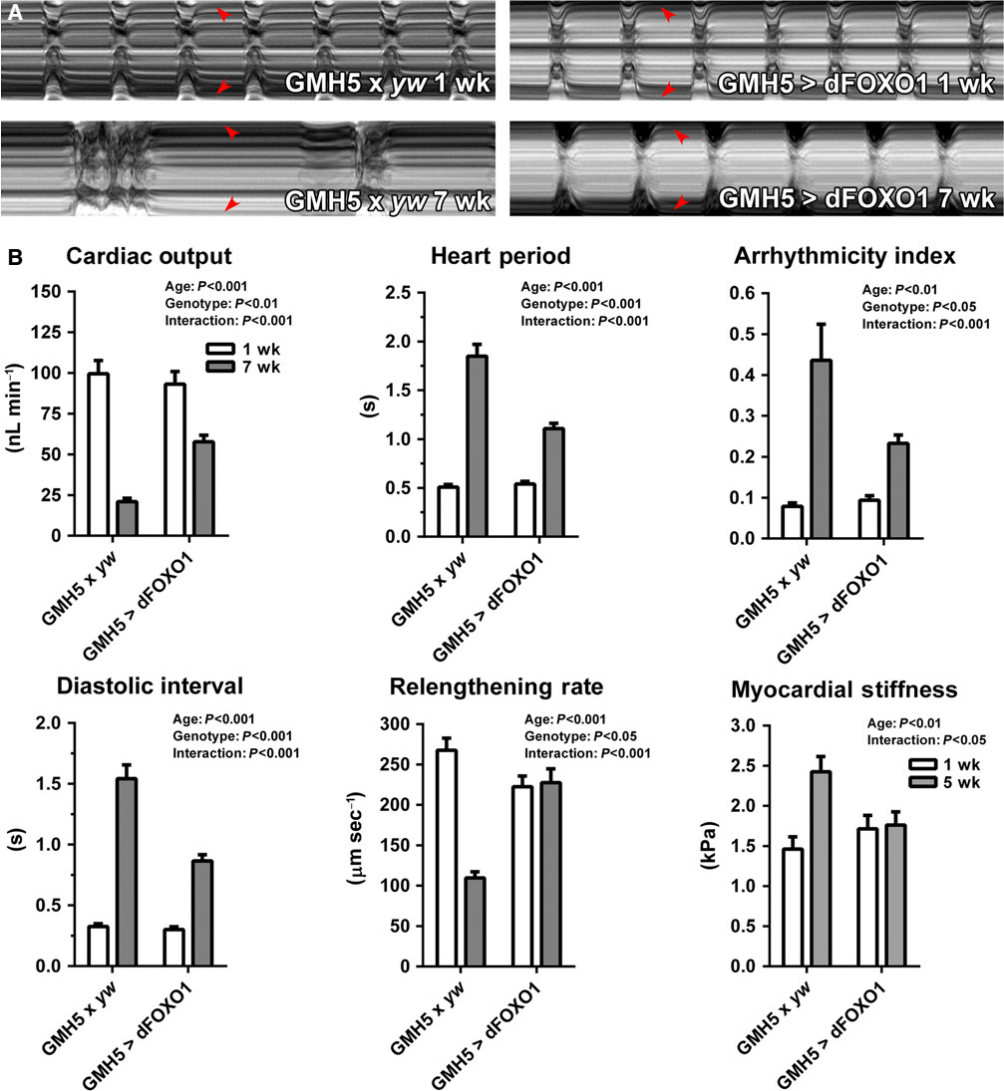
GMH5‐GAL4‐driven *dFOXO* overexpression ameliorates heart function decline in aging *Drosophila melanogaster*. (A). M‐mode kymographs of beating one‐week control and *dFOXO*‐overexpressing hearts demonstrate that both lines exhibited similar myocardial performance. At 7 weeks, dysfunction was apparent in control hearts, while *dFOXO*‐overexpressing hearts were protected. Red arrowheads delineate cardiac wall edges. (B) High‐speed video microscopy and motion analysis software (Cammarato *et al*., 2015) were used to quantitatively analyze beating *D. melanogaster* hearts at 1 and 7 weeks. Myogenic cardiac output was significantly reduced; heart period, arrhythmicity index, and diastolic interval were significantly elevated; and myocardial relengthening rate was significantly reduced in aged relative to young control flies. These age‐dependent changes in cardiac performance were ameliorated in the hearts of *dFOXO*‐overexpressing flies (*n* = 46–72, two‐way ANOVA, Bonferroni post‐hoc test). Transverse stiffness at the CC ventral midline was measured via AFM‐based nanoindentation (Kaushik *et al*., 2015). *dFOXO* overexpression eliminated stiffening normally observed from 1 through 5 weeks in control hearts (*n* = 24–31, two‐way ANOVA, Bonferroni post‐hoc test).

Decreased heart performance accompanies aging in mammals (Sheydina *et al*., 2011; Dai *et al*., 2012; Strait & Lakatta, 2012). Semi‐intact *D. melanogaster* heart preparations (Fig. 1B) were assessed via high‐speed video microscopy and motion analysis (Cammarato *et al*., 2015) to determine whether the dorsal vessel recapitulates specific hallmarks of age‐associated decline. Representative M‐mode kymographs show age‐related perturbations in cardiac wall movement in *GMH5‐GAL4 x yw* control flies (Fig. 2A). Specifically, between 1 and 7 weeks of age, control hearts experienced a highly significant decrease in cardiac output (100 ± 8 nL min^−1^ vs. 21 ± 2 nL min^−1^) (Fig. 2B). Additionally, they exhibited significant increases in heart period (0.5 ± 0.1 s vs. 1.9 ± 0.1 s), arrhythmicity index (0.08 ± 0.01 vs. 0.44 ± 0.09), and diastolic interval (0.3 ± 0.1 s vs. 1.5 ± 0.1 s) and a reduced myocardial relengthening rate (270 ± 20 μm s^−1^ vs. 110 ± 10 μm s^−1^). Age‐matched flies in which *dFOXO* was overexpressed exclusively in cardiomyocytes via GMH5‐GAL4 exhibited changes in cardiac output (93 ± 8 nL min^−1^ vs. 58 ± 4 nL min^−1^), heart period (0.5 ± 0.1 s vs. 1.1 ± 0.1 s), arrhythmicity index (0.09 ± 0.01 vs. 0.23 ± 0.02), diastolic interval (0.3 ± 0.1 s vs. 0.9 ± 0.1 s), and myocardial relengthening rate (220 ± 10 μm s^−1^ vs. 230 ± 20 μm s^−1^) that were significantly attenuated compared to controls. This pattern of improved performance was also observed at 5 weeks of age, which highlights dFOXO‐mediated cardioprotection against the progressive nature of functional decline throughout adult life (Fig. S3). The 1‐ and 7‐week results were corroborated using *GMH5‐GAL4 *>*  UAS‐dFOXO* line 2 (Fig. S4). No significant differences in cardiac physiology were observed between the two *GMH5‐GAL4 *>* UAS‐dFOXO* lines over time.

Mammalian hearts undergo myocardial stiffening with age (Sheydina *et al*., 2011; Dai *et al*., 2012; Strait & Lakatta, 2012). Likewise, *D. melanogaster* hearts display changes in passive mechanical properties over time (Kaushik *et al*., 2012, 2015). Using an atomic force microscopy (AFM)‐based approach, 5‐week control hearts were found to be significantly stiffer than 1‐week hearts (2.4 ± 0.2 kPa vs. 1.5 ± 0.2 kPa) (Fig. 2B). When *dFOXO* was overexpressed exclusively in the myocardium of age‐matched flies, however, the increase in stiffness was completely eliminated (1.8 ± 0.2 kPa vs. 1.7 ± 0.2 kPa). This finding, combined with the tempering of physiological decline in *dFOXO*‐overexpressing hearts, suggests that dFOXO is an important mediator of cardiomyocyte function and when overexpressed may decelerate normal cardiac aging.

### GMH5‐GAL4‐mediated *dFOXO* overexpression enhances UPS gene transcription and protein quality control in cardiomyocytes with age

**Figure 3:**
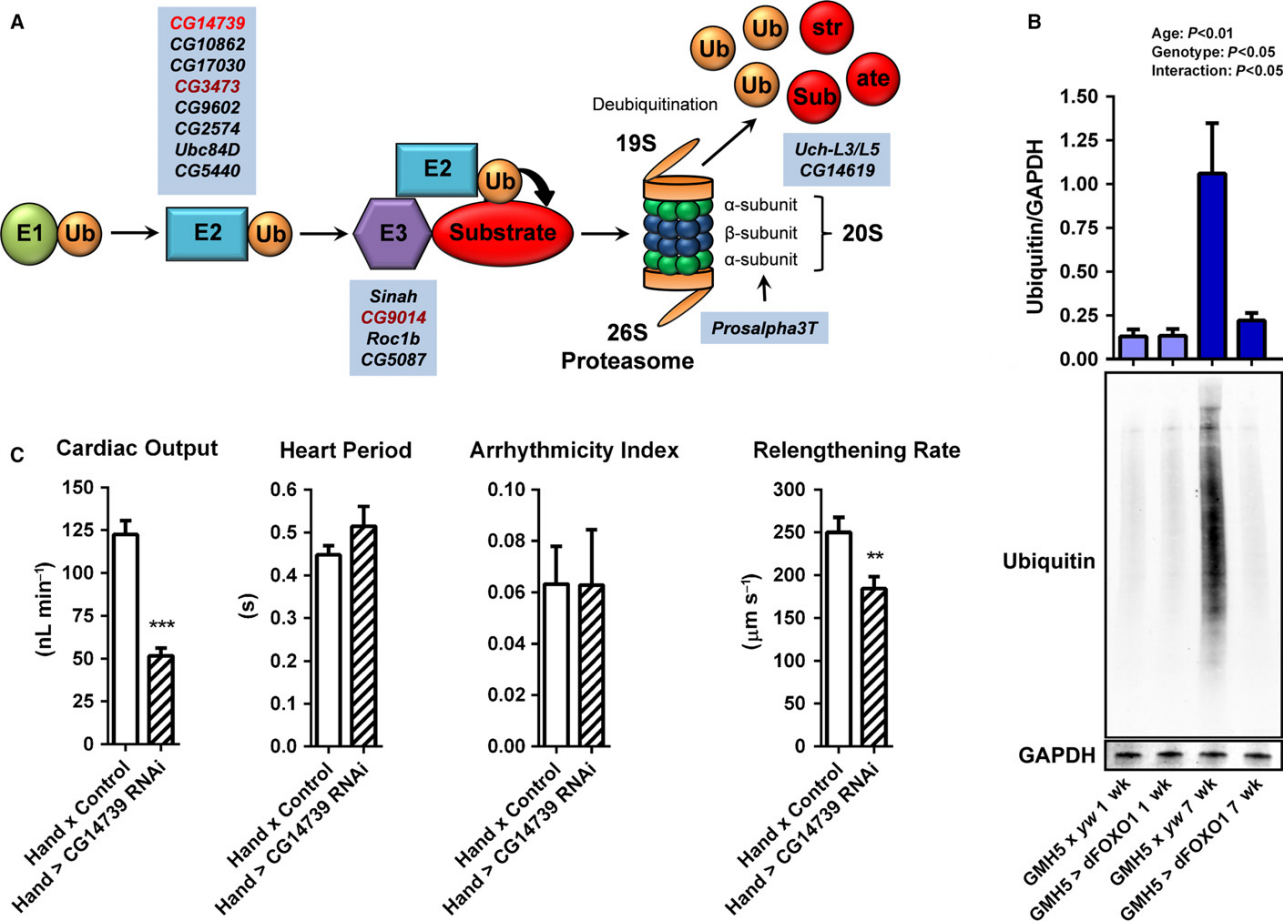
Cardiac‐specific *dFOXO* overexpression via GMH5‐GAL4 influences the transcription of genes associated with the UPS and reduces ubiquitinated protein content in aged fly hearts. (A) Illustration of the UPS. Ubiquitination of misfolded proteins occurs through a cascade of enzymatic reactions; the E1 ubiquitin activating enzyme transfers activated ubiquitin (Ub) to the E2 ubiquitin conjugating ligase, which then transfers Ub to the substrate, mediated through the E3 ubiquitin ligase. The ubiquitinated substrate is recognized, bound, and degraded by the 26S proteasome, which is composed of the 19S and 20S subunits. Highlighted in blue are fly homologs of UPS‐associated genes whose transcripts were downregulated in control hearts with age and upregulated in *dFOXO*‐overexpressing hearts (fold change ≥2.0, *P* < 0.05; Table S1) as determined via microarray analysis (Fig. S5). Upon cardiac‐restricted RNAi‐induced knockdown, the UPS gene in light red induced a premature aging myocardial phenotype, while those in dark red reduced lifespan (Neely *et al*., 2010). (B) Quantitative western blot analysis was used to probe 1‐ and 7‐week hearts for ubiquitin and GAPDH (loading control). At 7 weeks, control (*GMH5 x yw*) hearts accumulated a significant amount of ubiquitinated proteins compared to young hearts. Cardiac‐specific overexpression of *dFOXO* significantly reduced ubiquitinated protein content in 7‐week hearts compared to controls (*n* = 7–9, two‐way ANOVA, Bonferroni post‐hoc test). (C) *CG14739*, a conserved E2 ubiquitin conjugating enzyme (Fig. S7), was highly reduced in aged control hearts, whereas *dFOXO* overexpression caused the transcript's abundance to increase. Cardiac‐specific knockdown of *CG14739* significantly reduced output and myocardial relengthening rate (*n* = 26, **P <* *0.01, ***P <* *0.001, Student's *t*‐test) after only 1 week.

Advanced aging is characterized by progressive accumulation of cellular damage that is accompanied by decreased proteostasis (Sheydina *et al*., 2011; Dai *et al*., 2012; Strait & Lakatta, 2012). These changes are likely precipitated by altered expression of genes associated with major PQC processes. To determine if the mechanistic underpinnings of age‐associated changes in control hearts included transcriptional differences in specific PQC genes and whether dFOXO‐facilitated cardioprotection involved reversing such potential variations, Affymetrix microarray analysis was performed. We identified 2315 probesets that were differentially expressed (ANOVA *P *<* *0.05) among the groups (i.e. 1‐ and 5‐week control and 1‐ and 5‐week *GMH5‐GAL4* > *UAS‐dFOXO* hearts). These were clustered to identify prominent patterns of regulation across genotypes and with age (Fig. S5). Enrichment of clusters with Gene Ontology classifications revealed a number of biological functions that were either similarly (Fig. S5, Clusters 1 and 3) or differentially (Fig. S5, Clusters 2, 4–8) regulated by age between the two genotypes. This classification suggested that over time, the typical decline in UPS‐associated gene transcription was reversed in *dFOXO*‐overexpressing hearts (Fig. 3A, Table S1), and dFOXO‐mediated cardioprotection may be accompanied by enhanced proteostasis. These results were in contrast to previous reports that highlighted dFOXO‐driven alterations in the expression of genes associated with autophagy in skeletal muscle (Demontis & Perrimon, 2010), which in our myocardial array data exhibited minimal age‐associated or genotypic changes. Additionally, a number of transcripts from genes associated with the heat‐shock response (chaperone‐mediated protein refolding) similarly declined over time in control hearts, but were increased in 5‐week *dFOXO*‐overexpressing hearts. Finally, a bioinformatics approach verified that transgenic dFOXO actively regulated the expression of forkhead family gene targets in cardiomyocytes (Table S2).

Guided by the microarray results, which suggested a link between dFOXO and UPS‐associated gene transcription, we next tested whether improved cardiac performance in *dFOXO*‐overexpressing hearts was accompanied by reduced ubiquitinated protein content. Hearts from 1‐ and 7‐week control and *dFOXO*‐overexpressing flies were subjected to quantitative western blot analysis of nonspecific ubiquitinated proteins. Aging in controls was accompanied by significantly increased ubiquitinated protein content, as the resulting normalized anti‐ubiquitin signal was eight‐fold higher in aged compared to young hearts (1.09 ± 0.24 vs. 0.14 ± 0.02) (Fig. 3B). These results are consistent with previous findings that demonstrated an accumulation of ubiquitinated proteins in aged *D. melanogaster* flight muscle (Demontis & Perrimon, 2010; Rana *et al*., 2013). *dFOXO*‐overexpressing hearts had significantly reduced ubiquitin at 7 weeks compared to similarly aged controls (0.21 ± 0.05 vs. 1.09 ± 0.24), and there was no significant increase in 7‐ vs. 1‐week hearts (0.21 ± 0.05 vs. 0.15 ± 0.05). This was corroborated in aged *dFOXO*‐overexpressing hearts using *UAS‐dFOXO* line 2 (Fig. S6A). Indirect flight muscles (IFM) of 1‐ and 7‐week control and of cardiac‐specific *dFOXO*‐overexpressing flies were probed for ubiquitin (Fig. S6B). No difference in IFM ubiquitinated protein content was found, and therefore, *dFOXO* overexpression in the heart seemed to have no impact on systemic PQC. These data suggest that cardiac‐specific increases in dFOXO are accompanied by enhanced proteostasis locally and a concomitant amelioration of functional decline over time.

### Cardiac‐specific knockdown of UPS components disrupts heart performance in young flies

We previously conducted a genome‐wide RNAi screen to identify conserved genes that, when selectively suppressed in the *D. melanogaster* heart, impaired cardiac function as manifested by premature death following brief exposure to elevated temperature (Neely *et al*., 2010). Many components of the UPS were determined to be indispensable for heart performance in young flies including *CG9014* and *CG3473*, which when knocked down significantly reduced survival following stress. These E3 and E2 ligases (Fig. 3A) share 44% identity (~70% similarity) and 79% identity (~90% similarity) to human RNF41 and UBE2N, respectively (Fig. S7). According to our microarray data, *CG9014* and *CG3473* were downregulated with age in the hearts of control flies (−4.0‐fold change, FDR‐adjusted *P *<* *0.01; and −1.7‐fold change, FDR‐adjusted *P *<* *0.05, respectively) and exhibited augmented levels following GMH5‐GAL4‐mediated *dFOXO* overexpression (9.4‐fold change, FDR‐adjusted *P *<* *0.01, interaction *P *<* *0.001; and 9.3‐fold change, FDR‐adjusted *P *<* *0.01, interaction *P *<* *0.001, respectively). Likewise, *CG14739*, an E2 ligase that shares 42% identity (~60% similarity) with human UBE2H, was downregulated in control hearts with age (−18.9‐fold change, FDR‐adjusted *P *<* *0.001), but upregulated following *dFOXO* overexpression (13.9‐fold change, FDR‐adjusted *P *<* *0.01, interaction *P *<* *0.001). To test whether reductions in the E2 ligase can directly contribute to a decline in cardiac function, *CG14739* was knocked down in *D. melanogaster* hearts, which after 1 week showed a phenotype reminiscent of accelerated aging (Fig. 3C). Relative to controls, *CG14739 RNAi* hearts exhibited significantly decreased cardiac output (123 ± 8 nL min^−1^ vs. 52 ± 5 nL min^−1^) and myocardial relengthening rate (250 ± 20 μm s^−1^ vs. 180 ± 10 μm s^−1^).

### Extent of *dFOXO* overexpression can be controlled by drivers of differing strengths

**Figure 4:**
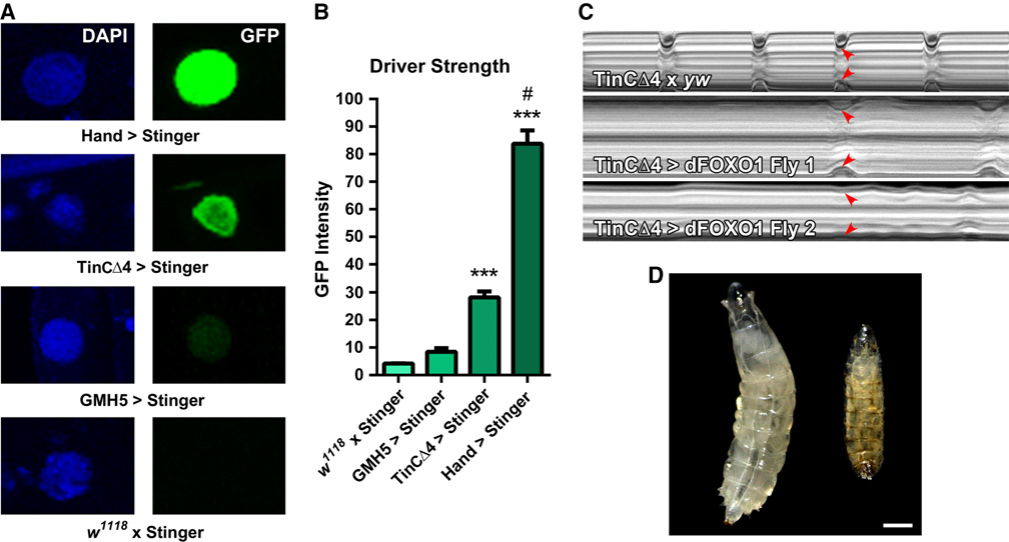
Excessive cardiac‐specific overexpression of *dFOXO* is detrimental to heart function and survival. (A) Relative driver strength was determined by crossing heart‐specific driver lines, *Hand* ^*4.2*^ *‐GAL4*,* TinCΔ4‐GAL4*, and *GMH5‐GAL4*, with *UAS‐Stinger*, a nuclear‐specific GFP reporter. Nuclear fluorescence from the progeny's cardiomyocytes was proportional to ‘driver strength’. (B) Emission intensities were quantified following uniform excitation for all genotypes and compared to control (*w* ^*1118*^ x *UAS‐Stinger*). Cardiomyocytes of *Hand* ^*4.2*^ *‐GAL4 > UAS‐Stinger* flies produced high nuclear fluorescence signals, while those from *TinCΔ4‐GAL4 >  UAS‐Stinger* flies produced moderate‐strength signals. Nuclei of *GMH5‐GAL4 > UAS‐Stinger* cardiomyocytes produced signals that were elevated, but at low excitation levels were not significantly different from control (*n* > 100 nuclei, ***P <* *0.001 compared to control and *GMH5 > Stinger*, #*P *<* *0.001 compared to *TincΔ4 > Stinger*, one‐way ANOVA, Bonferroni post‐hoc test). At high excitation, *GMH5‐GAL4 > UAS‐Stinger* cardiomyocytes produced signals that were elevated significantly vs. control (Fig. S8). (C) M‐mode kymographs of 1‐week control (top) and *TinCΔ4‐GAL4 > UAS‐dFOXO* line 1 hearts (middle and bottom) are displayed with systolic wall positions denoted by red arrow heads. Note poor, non‐rhythmic contractions characterized by lengthened diastolic and systolic intervals in the *dFOXO*‐overexpressing hearts. (D) Images of *Hand* ^*4.2*^ *‐GAL4* x *yw* control (left) and *Hand* ^*4.2*^ *‐GAL4 > UAS‐dFOXO* line 1 (right) 4‐day larvae. Control larvae developed to the third‐instar phase, while *Hand* ^*4.2*^ *‐GAL4 > UAS‐dFOXO* line 1 larvae typically developed to the second‐instar phase, ceased development, and died. Scale bar = 0.5 mm.

GMH5‐GAL4‐directed *dFOXO* overexpression ameliorated the functional decline of and improved proteostasis in aged *D. melanogaster* myocardium. However, FOXO transcription factors influence a host of cellular processes that, depending on the extent of activation, can induce seemingly contradictory adaptive or maladaptive effects (Rothermel & Hill, 2008; Maiese *et al*., 2009; Ferdous *et al*., 2010). Thus, to investigate potentially unique dose‐dependent responses to discrete quantities of *dFOXO* overexpression, several different cardiac‐specific drivers were employed. We first determined the relative expression strengths dictated by GMH5‐GAL4, TinCΔ4‐GAL4, and Hand^4.2^‐GAL4 by crossing each driver line with flies harboring *UAS‐Stinger*, a gene encoding a nuclear‐specific GFP reporter. As a negative control, *UAS‐Stinger* was crossed with *w* ^*1118*^. Hearts of adult progeny were imaged via confocal microscopy (Fig. 4A), and fluorescence emission resulting from uniform excitation across genotypes was measured (Fig. 4B). *Hand* ^*4.2*^ *‐GAL4 > UAS‐Stinger* cardiomyocyte nuclei emitted significantly high fluorescent signals compared with controls (84 ± 5 AU vs. 4.2 ± 0.1 AU), representative of excessive driver strength. *TinCΔ4‐GAL4 > UAS‐Stinger* cardiac nuclei produced fluorescence signals that were somewhat mosaic in nature. The average GFP intensity was significantly higher than control (28 ± 2 AU vs. 4.2 ± 0.1 AU), but less than that emitted by *Hand* ^*4.2*^ *‐GAL4 > UAS‐Stinger* cardiac nuclei; therefore, TinCΔ4‐GAL4 was characterized as an intermediate‐strength heart‐specific driver. *GMH5‐GAL4 > UAS‐Stinger* cardiac nuclei emitted fluorescence that was often undetectable except in the conical chamber (CC) and ostia inflow tracts. This resulted in an average emission value that did not differ from control at low excitation intensity (8.5 ± 1.3 AU vs. 4.23 ± 0.05 AU). At a higher uniform laser intensity, however, the nuclear fluorescence signal from *GMH5‐GAL4 > UAS‐Stinger* cardiomyocytes was significantly greater than that from *UAS‐Stinger* x *w* ^*1118*^ (Fig. S8). These findings revealed that GMH5‐GAL4, although modest in overall strength on average, significantly overexpressed *UAS‐Stinger* in *D. melanogaster* cardiomyocytes, consistent with earlier *GMH5‐GAL4 > UAS‐dFOXO* mRNA quantification results (Fig. 1D).

### dFOXO dosage affects development and cardiac performance

Mild *dFOXO* overexpression via GMH5‐GAL4 benefitted *D. melanogaster* hearts with age. When *dFOXO* was overexpressed in the dorsal vessel using the intermediate‐strength TinCΔ4‐GAL4 driver (Figs S1 and S2), however, progeny developed more slowly than controls, adult flies lived an average of 2 weeks, and cardiac performance was severely compromised at 1 week (Fig. 4C). When *dFOXO* was overexpressed in the heart via the high‐strength Hand^4.2^‐GAL4 driver, progeny rarely developed beyond the second instar larval stage (Fig. 4D). These data imply that discrete levels of *dFOXO* expression can mediate distinct cardiac responses and that excessive doses of dFOXO, determined by the relatively strong TinCΔ4‐ or Hand^4.2^‐GAL4 drivers, do not provide further benefit over time and are, in fact, detrimental to heart function and survival.

**Figure 5:**
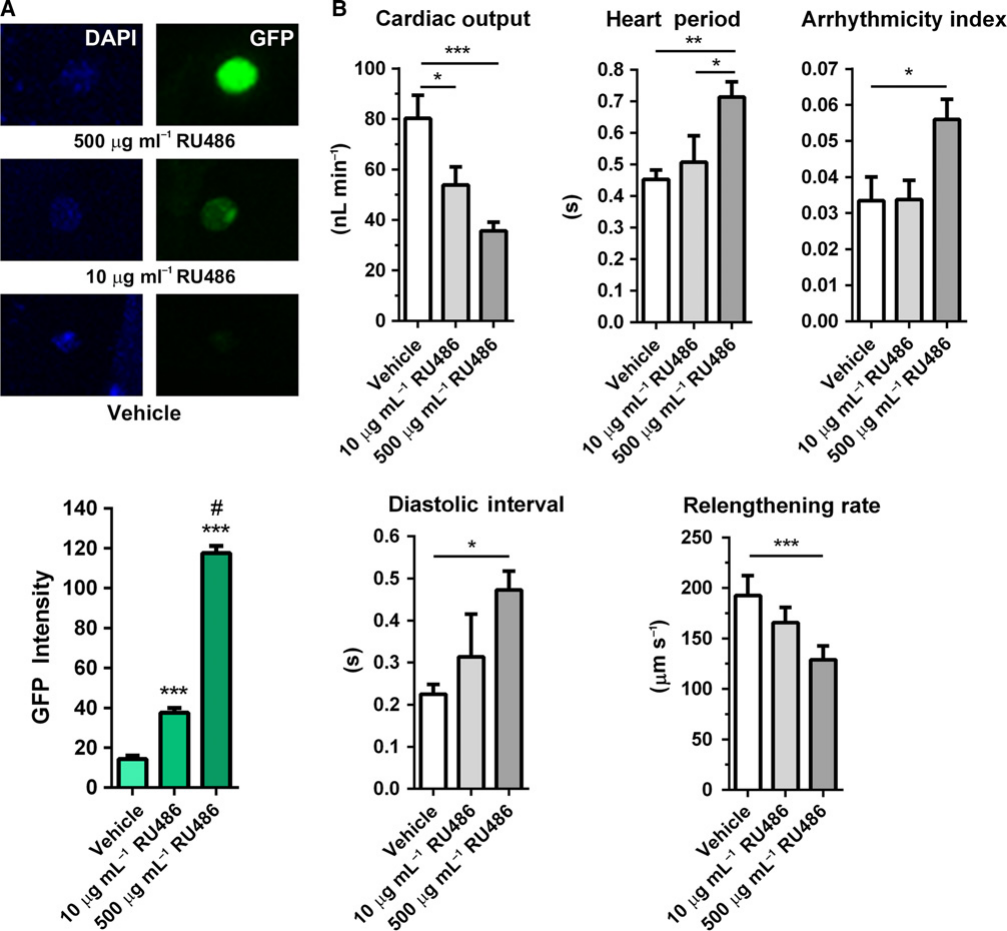
The maladaptive, cardiac‐specific dosing effect of *dFOXO* overexpression is evident post‐development. (A) To visualize and quantify dose‐dependent expression responses in adults, *Hand* ^*4.2*^ *‐GS‐GAL4* flies were crossed with *UAS‐Stinger* to produce progeny whose cardiomyocyte nuclei would fluoresce upon exposure to RU486 due to conditional *GFP* expression. Dose‐dependence was confirmed when vehicle, 10 μg mL^−1^, or 500 μg mL^−1^ RU486 was added to food at 2 days post‐eclosion and fly hearts imaged after 7 days (top). Graded nuclear emission intensities were observed following incubation with increasing amounts of RU486 (bottom; *n* > 100, ***P <* *0.001 compared with vehicle, #*P *<* *0.001 compared with 10 μg mL^−1^ RU486, one‐way ANOVA, Bonferroni post‐hoc test). (B) *Hand* ^*4.2*^ *‐GS‐GAL4* were crossed with *UAS‐dFOXO* line 1, and progeny were treated with vehicle, 10 μg mL^−1^, or 500 μg mL^−1^ RU486 at 2 days post‐eclosion. After 7 days, the hearts were analyzed as previously described. Flies exposed to the high dose of RU486 experienced significantly decreased cardiac output, elevated heart period, diastolic interval, and arrhythmicity index, and decreased myocardial relengthening rate compared to controls. Cardiac output was also significantly reduced in flies exposed to the low drug dose, but to a lesser extent than flies that consumed high doses of RU486 (*n* = 15, *P <* *0.05, **P <* *0.01, ***P <* *0.001, one‐way ANOVA, Bonferroni post‐hoc test).

To directly confirm dose‐dependent responses to *dFOXO* overexpression in adult hearts and, since TinCΔ4‐ and Hand^4.2^‐GAL4 are expressed during early cardiogenesis, to simultaneously eliminate confounding developmental variables, an RU486‐inducible GeneSwitch GAL4 driver was utilized in conjunction with the *Hand* ^*4.2*^ enhancer (*Hand* ^*4.2*^ *‐GS‐GAL4*) (Nicholson *et al*., 2008). To verify that the amount of cardiac‐specific *UAS‐transgene* expression was directly proportional to the dosage of RU486, *Hand* ^*4.2*^ *‐GS‐GAL4* flies were crossed with *UAS‐Stinger* flies. At 2 days post‐eclosion, adult progeny (*Hand* ^*4.2*^ *‐GS‐GAL4 > UAS‐Stinger*) were placed on food containing vehicle (100% EtOH), 10 μg mL^−1^ RU486, or 500 μg mL^−1^ RU486. After 7 days, hearts were exposed and visualized via confocal microscopy, and nuclear fluorescence was quantified as previously (Fig. 5A). Cardiomyocyte nuclei of flies on 500 μg mL^−1^ RU486 emitted the highest intensity signals (118 ± 4 AU), hearts of flies on 10μg mL^−1^ RU486 emitted less intense fluorescence (37 ± 3 AU), and those on vehicle produced very little fluorescence (14 ± 2 AU), assuring that the amount of *UAS‐*transgene expression was proportional to drug dosage.

*Hand* ^*4.2*^ *‐GS‐GAL4* flies were crossed with *UAS‐dFOXO* line 1, and the resulting adult progeny were subjected to food supplemented with vehicle, 10 μg mL^−1^ RU486, or 500 μg mL^−1^ RU486. After 7 days, low‐dose transgene activation resulted in a modest but significant reduction in cardiac output relative to controls (54 ± 7 nL min^−1^ vs. 80 ± 9 nL min^−1^) (Fig. 5B). However, other indices of heart performance did not significantly differ from those of flies feeding on vehicle. Compared to controls, flies feeding on food supplemented with 500 μg mL^−1^ RU486 experienced significantly decreased cardiac output (80 ± 9 nL min^−1^ vs. 36 ± 3 nL min^−1^), increased heart period (0.45 ± 0.03 s vs. 0.71 ± 0.05 s), arrhythmicity index (0.03 ± 0.01 vs. 0.06 ± 0.01), and diastolic interval (0.22 ± 0.02 s vs. 0.47 ± 0.05 s) and reduced myocardial relengthening rate (193 ± 20 μm s^−1^ vs. 129 ± 14 μm s^−1^). These data suggest that the severities of the maladaptive responses to *dFOXO* overexpression in the adult heart via TinCΔ4‐ and Hand^4.2^‐GAL4 result, at least in part, from excessive dFOXO doses rather than exclusively from developmental complications.

### Cardiac‐specific *dFOXO* knockdown is detrimental to heart performance in young flies

**Figure 6:**
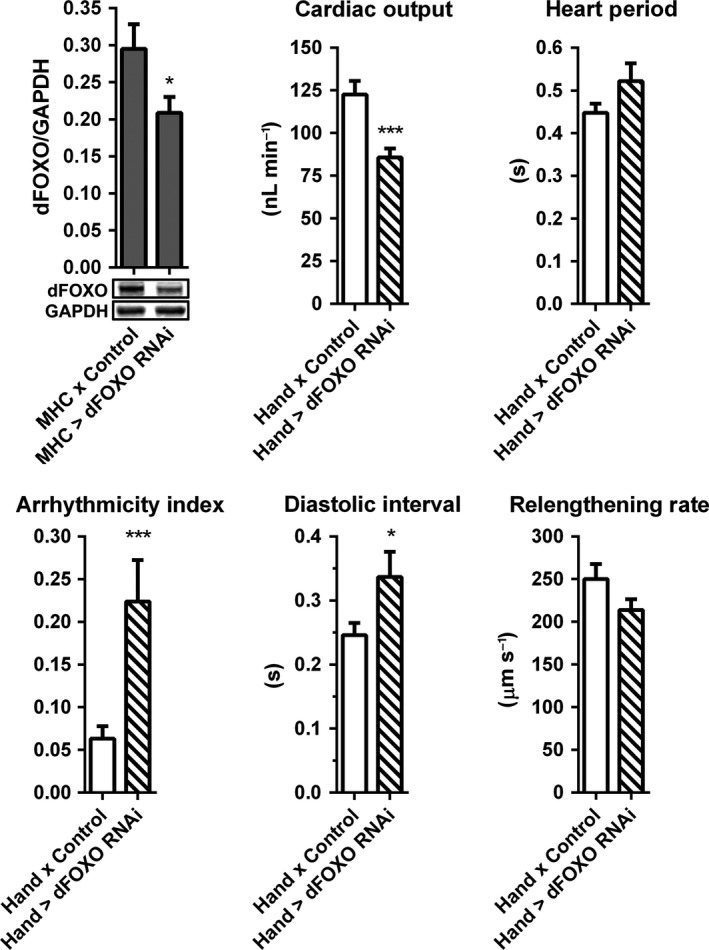
Knockdown of *dFOXO* perturbs cardiac performance in young flies. (A) *dFOXO*‐RNAi was expressed in all muscle via MHC‐GAL4. Reduced dFOXO protein content was confirmed by quantitative western blot analysis of whole thoraces (*n* = 16, *P <* *0.05, Student's *t*‐test). (B) At 1 week, relative to controls, *Hand* ^*4.2*^ *‐GAL4 *>* UAS‐dFOXO‐RNAi* flies exhibited a significant reduction in cardiac output and significant increases in arrhythmicity index and diastolic interval, indicating acute cardiac dysfunction (*n* = 26–29, *P <* *0.05, ***P <* *0.001, Student's *t*‐test).

To determine if suppressing *dFOXO* specifically in the heart had a similarly negative impact on performance, a UAS‐controlled transgenic RNAi line targeting *dFOXO* was obtained. To confirm the efficacy of dFOXO knockdown, the interfering RNA was first expressed in skeletal muscle using MHC‐GAL4. The dFOXO/GAPDH level in thoracic musculature of *dFOXO‐RNAi*‐expressing flies was reduced by ~30% compared to controls (dFOXO/GAPDH = 0.20 ± 0.02 vs. 0.28 ± 0.03) (Fig. 6A). *UAS‐dFOXO‐RNAi* flies were crossed with *Hand* ^*4.2*^ *‐GAL4* flies, and heart performance of adult progeny was analyzed at 1 week (Fig. 6B). Many of the differences between control and *dFOXO‐RNAi*‐expressing hearts, including decreased cardiac output (123 ± 8 nL min^−1^ vs. 86 ± 5 nL min^−1^), increased incidence of arrhythmia (0.06 ± 0.01 vs. 0.22 ± 0.05), and increased diastolic interval (0.25 ± 0.02 s vs. 0.34 ± 0.04 s), mirrored the effects of aging. Thus, a loss of *dFOXO* causes cardiac dysfunction acutely.

## Discussion

Here, we sought to define the spectrum of physiological changes associated with cardiac autonomous manipulation of *FOXO* expression, *in vivo*, and the molecular mechanisms behind FOXO‐mediated protection from the inevitable decline of heart performance. *D. melanogaster* is an elegant model for studying cardiac senescence. Within weeks, its heart experiences decreased cardiac output, higher incidence of arrhythmia, prolonged diastolic periods, reduced myocardial relengthening rates, and increased stiffness (Fig. 2B). Cardiac output, heart period, and arrhythmicity index reflect overall pumping ability. Interestingly, mild increases in dFOXO significantly attenuated the progressive changes normally observed in these functional indices. Diastolic interval, myocardial relengthening rate, and stiffness are useful for gauging diastolic performance and, when altered, can indicate diastolic dysfunction (Borbely *et al*., 2009; Dai *et al*., 2012; Kaushik *et al*., 2012; Strait & Lakatta, 2012). Moderate *dFOXO* overexpression also mitigated changes in these parameters and, hence, suppressed diastolic dysfunction. Age‐associated diastolic dysfunction may be caused by impaired calcium handling and signaling, increased deposition and cross‐linking of the extracellular matrix, accumulation of protein aggregates, and/or abnormalities in myofilamentous and cytoarchitectural components (Bers, 2002; Borbely *et al*., 2009; Kazik *et al*., 2010; Sheydina *et al*., 2011; Strait & Lakatta, 2012). FOXOs can directly and indirectly regulate the expression of genes involved with these cellular components and processes (Sheydina *et al*., 2011; Bai *et al*., 2013), a potential means by which symptoms of dysfunction are alleviated. For example, our transcriptomic data revealed several relevant genes that were differentially expressed in aged *dFOXO*‐overexpressing hearts compared to control hearts including homologs of the human ryanodine receptor, SERCA, PI3K, IGF‐I, ERK2, collagens, tropomyosin, actins, α‐actinin, and tropomodulin. Moreover, since FOXOs are involved in mediating the expression of an array of genes associated with proteostasis, mild overexpression could also assist in improving the turnover of proteins associated with calcium handling, systolic and diastolic mechanics, and the cytostructure.

In vertebrate cardiomyocytes, disease‐associated changes in proteostasis are well‐documented. Our microarray results illustrate that over time, in healthy flies, expression of genes associated with the UPS and chaperone‐mediated protein refolding normally declines. In 5‐week control hearts, we identified reduced transcripts from genes involved in all steps along the UPS pathway (Fig. 3A, Table S1), the role of which is decidedly underreported in the context of cardiac aging (Quarles *et al*., 2015). These changes were reversed in aged *GMH5‐GAL4 >  UAS‐dFOXO* hearts (Table S1, Fig. S5). This implies that modest *dFOXO* overexpression mediates improved cardiac aging, in part, by modulating the entire pathway. Previous results showed that cardiac‐specific RNAi‐mediated knockdown of the E3 ubiquitin ligase *CG9014* and the E2 ubiquitin conjugating enzyme *CG3473*, homologs of human RNF41 and UBE2N, respectively, increased the susceptibility for premature death (Neely *et al*., 2010). We found that these genes were, in fact, significantly downregulated in aged control hearts (Fig. 3A). Reduced activity of such genes may substantially increase the number of misfolded proteins not labeled for degradation, leading to proteotoxicity, and subsequently impact cardiac performance (Mearini *et al*., 2008; Gupta *et al*., 2014). RNAi‐mediated knockdown of *CG14739*, an E2 ubiquitin conjugating enzyme that was also highly downregulated in aged hearts, triggered cardiac dysfunction after only 1 week, indicative of premature aging (Fig. 3C). Expression of genes associated with proteasomal degradation and deubiquitination, *Prosalpha3T* and *Uch‐L3*/*L5* (Fig. 3A), was also substantially reduced in old control (−1.6‐fold change, FDR‐adjusted *P *<* *0.05; and −25.6‐fold change, FDR‐adjusted *P *<* *0.001, respectively) and augmented in *dFOXO*‐overexpressing hearts (17.8‐fold change, FDR‐adjusted *P *<* *0.01, interaction *P *<* *0.001; and 19.0‐fold change, FDR‐adjusted *P *<* *0.01, interaction *P *<* *0.001, respectively), potentially enhancing ubiquitinated protein removal and recycling (Fig. 3B). Additionally, particular genes associated with chaperone‐mediated protein refolding, such as the *D. melanogaster* homologs of Hsp70 (*Hsp67Bc*) and αB crystallin (*Hsp27*), were downregulated in control (−1.1‐fold change, FDR‐adjusted *P *=* *0.35; and −1.1‐fold change, FDR‐adjusted *P *=* *0.33, respectively), but upregulated in *dFOXO*‐overexpressing hearts over time (2.3‐fold change, FDR‐adjusted *P *<* *0.05, interaction *P *<* *0.05; and 1.2‐fold change, FDR‐adjusted *P *=* *0.44, interaction *P *<* *0.05, respectively). Similar changes in chaperone gene expression were observed in aged *dFOXO*‐overexpressing flight muscle (Demontis & Perrimon, 2010). By bolstering transcription of genes associated with the UPS and chaperone‐mediated protein refolding and reducing the number of ubiquitinated proteins (Fig. 3B), mild *dFOXO* overexpression alleviates age‐associated cardiac proteotoxicity. Importantly, our microarray data additionally highlight several other differentially regulated genes that may also contribute to dFOXO‐directed improvements in heart function during nonpathological aging (Table S3).

Current and previous findings imply that FOXO may serve as a viable therapeutic option to alleviate age‐associated cardiac deterioration. However, as demonstrated here, manipulating *dFOXO* expression adversely influenced cardiac function and homeostasis in most cases (Figs 4C,D and 5B). These results are consistent with graded activity levels of FOXO‐mediated PQC processes that can elicit diverse functional consequences, as formerly suggested. For example, based on several lines of *in vitro* and *in vivo* evidence, Ferdous *et al*. (2010) proposed that the extent of FOXO‐regulated autophagic activity can have either protective or destructive effects in myocardia. Excessive amounts of *FOXO* expression in isolated cardiomyocytes produce maladaptive effects associated with rampant autophagy (Zhu *et al*., 2007; Rothermel & Hill, 2008; Ferdous *et al*., 2010). Conversely, too little *FOXO* expression, and thus impaired proteostasis, can negatively affect function by promoting accumulation of misfolded protein aggregates and dysfunctional organelles (Ferdous *et al*., 2010). Additional evidence suggests that UPS activity can have a positive or negative impact on cardiomyocytes in the context of disease (Mearini *et al*., 2008; Sengupta *et al*., 2011), which may be further influenced by FOXO activity. Here, *dFOXO* expressed just above endogenous levels via GMH5‐GAL4 provided a cardioprotective age‐associated response, consistent with enhanced PQC (Fig. S9). However, heart function of flies in which a ‘low dose’ of RU486 was utilized to drive *dFOXO* appeared slightly hampered, likely because the dosage exceeded that dictated by GMH5‐GAL4. Following excessive *dFOXO* expression, initiated by ‘high doses’ of RU486, TinCΔ4‐GAL4, or Hand^4.2^‐GAL4 drivers, remarkably poor cardiac performance and even organismal death were evident, which are in agreement with maladaptive consequences including excessive catabolism, autophagic cell death, and apoptosis. Reduced cardiac performance was also observed in young flies expressing *dFOXO‐RNAi*, consistent with insufficient PQC. These results, obtained in a single model system, provide direct support of dose‐dependent adaptive vs. maladaptive effects following cardiac‐specific *dFOXO* overexpression or knockdown. Thus, if FOXO were to be used as a therapy against age‐associated cardiac decline, its dosage would need to be tightly regulated.

Advanced age is accompanied by impaired cardiac function and, in general, by reduced PQC (Lopez‐Otin *et al*., 2013). The decline of UPS activity, autophagy, and chaperone‐mediated protein refolding is exceptionally detrimental to cell types that are postmitotic and exhibit limited regenerative capacity. Here, we show that slightly elevated expression of *dFOXO* in *D. melanogaster* cardiomyocytes alleviates the persistent deterioration of heart performance. While there is an increasing appreciation for interplay between the UPS and autophagy in the heart (Wang & Robbins, 2014; Wang & Wang, 2015), our data suggest that dFOXO, when appropriately titered, affords age‐associated cardioprotection through enhanced UPS activation vs. autophagy. This distinguishes the current findings from those obtained in skeletal muscle (Demontis & Perrimon, 2010) and supports the conditional use of distinct FOXO‐mediated proteostatic mechanisms among the tissue types. Moreover, our results illustrate that the myocardium is particularly sensitive to both excessive and insufficient dFOXO doses, both of which engendered severely maladaptive responses. Negative dose responses were also identified in other *dFOXO*‐overexpressing tissues, including skeletal muscles (Table S4), which previously went unreported. Such responses likely preclude FOXO as a viable therapeutic modality. Nonetheless, detailed examination of microarray data from modest *dFOXO*‐overexpressing hearts highlights downstream targets that, when selectively overexpressed, could bypass the negative dose‐dependent effects of dFOXO itself (Tables S1 and S3). Overall, our findings highlight the utility of *D. melanogaster* as an ideal model for exploring cardiac aging and for investigating novel therapeutic targets, including individual components of the UPS and chaperone‐mediated protein refolding, that may assist in preserving cardiac homeostasis and performance in the elderly.

## Experimental procedures

Detailed descriptions of experimental procedures, reagents, and associated references can be found in online supporting information.

### *Drosophila melanogaster* strains

Flies were raised on standard medium at 25 °C. *w* ^*1118*^, *UAS‐FOXO* line 1 (*yw*;; *UAS‐FOXO wt.m3‐1*), *UAS‐Stinger*,* Hand* ^*4.2*^ *‐GS‐GAL4*,* Hand* ^*4.2*^ *‐GAL4*,* TinCΔ4‐GAL4*,* GMH5‐GAL4*, and *MHC‐GAL4 D. melanogaster* were employed for most studies. *UAS‐FOXO* lines 2 and 3 (*yw; P{UAS‐foxo.P}2* and *w* ^*1118*^ *; P{UASp‐foxo.S}3*) and *yw* flies were obtained from the Bloomington *Drosophila* Stock Center. Transgenic RNAi lines and appropriate controls were acquired from the Vienna *Drosophila* RNAi Center. Crosses were conducted by mating virgin *GAL4* females with young *UAS‐transgene* males. All experiments were performed on female flies collected no more than 8 hours post‐eclosion.

For GeneSwitch (GS) experiments, flies were placed on standard media immediately after eclosion. At 2 days of age, they were placed on media topped with vehicle (100% ethanol) or with RU486 diluted to various doses in 100% ethanol. Flies were transferred each day onto new food with drug or vehicle for 7 days.

### Heart tube analysis

Semi‐intact *D. melanogaster* heart tubes (Fig. 1B) were prepared and cardiac performance evaluated according to published procedures (Vogler & Ocorr, 2009; Cammarato *et al*., 2015; Kaushik *et al*., 2015). Significance was determined as below.

Myogenic ‘cardiac output’ was calculated as follows. Individual frames of hearts during peak diastole and systole were obtained from high‐speed videos. A two‐dimensional area was determined for a specific length (L), which generally encompassed abdominal segments two through four of the heart tubes (Fig. 1B) for systole and diastole, and from this an average diameter for the segment of each heart was ascertained. This segment was modeled as a cylinder. πr^2^·L was used to determine average systolic and diastolic volumes over the designated length to provide a ‘stroke volume’ (diastolic–systolic volume). Cardiac output (nL s^−1^) was calculated as stroke volume ∙ heart rate. Significance was determined as below.

AFM‐based nanoindentation, to determine transverse stiffness of the CC (Fig. 1B), was performed as described previously (Kaushik *et al*., 2015). The 5‐week time point was chosen since the procedure for exposing the CC for AFM is delicate, and as flies age beyond 5 weeks, the risk of tissue injury is elevated. Significance was determined as below.

### Quantitation of protein content

To quantify dFOXO, whole thoraces (two per biological replicate) were homogenized in Laemmli sample buffer and subjected to SDS‐PAGE, transferred to nitrocellulose, and probed with rabbit anti‐dFOXO and goat anti‐GAPDH antibodies. After incubation in IRDye secondary antibodies, the membranes were scanned using the Odyssey Infrared Imager and analyzed via Odyssey Application software. Mean intensity values for dFOXO from 8 to 16 biological replicates with two to three technical replicates each were normalized to GAPDH signals. Significant differences were assessed as below.

Ubiquitinated protein content was quantified from isolated hearts (6 to 10 per biological replicate) and evaluated as above using primary mouse anti‐ubiquitin and goat anti‐GAPDH antibodies. Mean intensity values for ubiquitin from three to nine biological replicates with at least two technical replicates each were normalized to GAPDH signals. Significant differences were determined as below.

### Fluorescence RNA *in situ* hybridization

To quantitate *dFOXO* transcripts, hearts were dissected and exposed (Fig. 1B) and contractions arrested using 10 mM EGTA. Samples were fixed in 4% paraformaldehyde for 30 minutes, washed three times in PBST, and stored in a 96‐well plate in PBS at 4 °C overnight. *In situ* hybridization was performed as reported previously (Viswanathan *et al*., 2016) using the QuantiGene^®^ ViewRNA^™^ Cell Assay kit from Panomics according to the manufacturer's suggested protocol with sequence specific probes designed to detect *dFOXO* and *GAPDH* mRNA (catalog numbers VF1‐18189 and VF6‐18191, respectively). Hearts were visualized with a Leica TCS SPE RGBV confocal microscope at 40X. Care was taken not to record the signal from non‐cardiac cells.

For quantitation of transcripts from confocal micrographs, channels were separated and colors converted to grayscale. Images were opened in ImageJ, changed to 8‐bit, and the threshold was adjusted to an upper limit of 150 and a lower limit of 55. Each region of interest was outlined and the number of particles, which represented *dFOXO* or *GAPDH* messages, determined. *dFOXO* was normalized to *GAPDH* particle number for cardiomyocytes from each of 16–20 hearts per genotype. Significance was assessed as below.

### Imaging of *Drosophila melanogaster* cardiomyocyte nuclei and larvae

Hearts of flies expressing *UAS‐stinger*, a nuclear GFP, were surgically exposed and contractions arrested using 10 mM EGTA. Samples were fixed, washed, and mounted on glass slides in ProLong^®^ Gold antifade reagent with DAPI, and imaged by confocal microscopy at 40X. Nuclear fluorescence intensity was quantified using Leica LAS AF software. All hearts per experiment were visualized once over the period of 1 day to avoid differences in laser intensity and photobleaching. Significant differences were calculated as below.

*Hand* ^*4.2*^ *‐GAL4* was crossed with *UAS‐dFOXO* line 1 or *yw* control. At 4 days post‐fertilization, larvae from both crosses were imaged with a Leica M165FC microscope fitted with a Leica EC3 camera.

### Statistical analyses

All statistical analyses were performed using GraphPad Prism 5.01. Values were determined to be normally distributed by post‐hoc normality tests. Significant differences between genotype and age, and interaction effects, were determined using two‐way analysis of variance (ANOVA) with Bonferroni post‐hoc tests. In experiments in which different ages were not investigated, one‐way ANOVA or Student's *t*‐tests were used to determine significance. Significance was reported at *P *<* *0.05. Pooled data are represented as mean ± SEM. All experiments were performed with the indicated sample sizes.

## Author contributions

ACB‐B, AJE, RB, and AC designed the studies. ACB‐B, ACZ, GK, MCV, and AC conducted the experiments. ACZ performed microarray and bioinformatics analysis. ACB‐B, ACZ, GK, AJE, and AC analyzed the data. ACB‐B, ACZ, GK, MCV, AJE, RB, and AC wrote, edited, and approved the submitted version of the manuscript.

## Funding

This work was supported by grants from the NIH (5T32HL007227‐38 to ACB‐B, NIHHL098053‐04 to ACZ, R01AG045428 to AE, T32HL105373 to GK, P01HL098053 and P01AG033561 to RB, and 1R56HL124091‐01 and 1R01HL124091‐01 to AC), AHA (10SDG2630130 to ACZ, 13PRE14410037 to GK, and 10SDG4180089 to AC), the Ellison Medical Foundation (to RB), and the American Federation for Aging Research (to AC).

## Conflict of interest

None declared.

## Supporting information


**Appendix S1** Experimental procedures.
**Fig. S1** *dFOXO* transcripts are more abundant in hearts overexpressing transgenic *dFOXO* compared to control.
**Fig. S2** Immunolocalization of dFOXO following overexpression via GMH5‐ or TinCΔ4‐GAL4.
**Fig. S3** GMH5‐GAL4‐ driven *dFOXO* overexpression suppresses heart function decline in aging *D. melanogaster*.
**Fig. S4** Amelioration of functional decline in aging *dFOXO*‐overexpressing hearts is corroborated with a second *UAS‐dFOXO* transgenic line.
**Fig. S5** Cardiac‐specific *dFOXO* overexpression via GMH5‐GAL4 influences the transcription of genes associated with many biological functions and components including the UPS.
**Fig. S6** GMH5‐GAL4‐driven *dFOXO* overexpression in lines 1 and 2 reduces ubiquitinated protein in aged fly hearts, while ubiquitin content in aged IFMs is not affected.
**Fig. S7** Sequence alignments of UPS‐associated proteins.
**Fig. S8** The GMH5‐GAL4 driver overexpresses UAS‐controlled transgenes in cardiomyocytes above endogenous levels.
**Fig. S9** Discrete quantities of dFOXO potentially result in graded levels of PQC activity that can positively or negatively affect the heart.
**Table S1** UPS‐associated genes whose transcription is significantly altered in hearts with modest *dFOXO* overexpression compared to control hearts.
**Table S2** Enrichment of evolutionarily conserved forkhead binding sites.
**Table S3** Additional pathways and genes that are differentially regulated with age between *GMH5‐GAL4* x *yw* vs. *dFOXO*‐overexpressing hearts that may contribute to dFOXO‐directed improvements in heart function during non‐pathological aging.
**Table S4** Cardiac and developmental consequences resulting from combinations of GAL4 drivers with various *UAS‐dFOXO* overexpression and knockdown constructs.
