# Supplementary material for: Modest overexpression of FOXO maintains cardiac proteostasis and ameliorates age‐associated functional decline
Source: Aging Cell. 2017 Jan 16;16(1):93–103. doi: 10.1111/acel.12543 (PMC5242305; doi:10.1111/acel.12543)
Supplement: Supplementary file 1 — Appendix S1 Experimental procedures. Fig. S1 dFOXO transcripts are more abundant in hearts overexpressing transgenic dFOXO compared to control. Fig. S2 Immunolocalization of dFOXO following overexpression via GMH5‐ or TinCΔ4‐GAL4. Fig. S3 GMH5‐GAL4‐ driven dFOXO overexpression suppresses heart function decline in aging D. melanogaster. Fig. S4 Amelioration of functional decline in aging dFOXO‐overexpressing hearts is corroborated with a second UAS‐dFOXO transgenic line. Fig. S5 Cardiac‐specific dFOXO overexpression via GMH5‐GAL4 influences the transcription of genes associated with many biological functions and components including the UPS. Fig. S6 GMH5‐GAL4‐driven dFOXO overexpression in lines 1 and 2 reduces ubiquitinated protein in aged fly hearts, while ubiquitin content in aged IFMs is not affected. Fig. S7 Sequence alignments of UPS‐associated proteins. Fig. S8 The GMH5‐GAL4 driver overexpresses UAS‐controlled transgenes in cardiomyocytes above endogenous levels. Fig. S9 Discrete quantities of dFOXO potentially result in graded levels of PQC activity that can positively or negatively affect the heart. Table S1 UPS‐associated genes whose transcription is significantly altered in hearts with modest dFOXO overexpression compared to control hearts. Table S2 Enrichment of evolutionarily conserved forkhead binding sites. Table S3 Additional pathways and genes that are differentially regulated with age between GMH5‐GAL4 x yw vs. dFOXO‐overexpressing hearts that may contribute to dFOXO‐directed improvements in heart function during non‐pathological aging. Table S4 Cardiac and developmental consequences resulting from combinations of GAL4 drivers with various UAS‐dFOXO overexpression and knockdown constructs. [file ACEL-16-93-s001.docx]

**Supplemental Experimental Procedures**

*D. melanogaster strains*

Flies were raised on standard cornmeal, agar, and molasses medium at 25°C with normal light/dark cycles. *w^1118^*, *UAS-FOXO* line 1 (*yw*;; *UAS-FOXO wt.m3-1*) (Wessells et al. 2004), *UAS-Stinger* (Barolo et al. 2000), *Hand^4.2^-GS-GAL4* (Monnier et al. 2012), *Hand^4.2^-GAL4* (Han and Olson 2005), *TinCΔ4-GAL4* (Yin et al. 1997), *GMH5-GAL4* (Wessells et al. 2004), *UH3-GAL4* (Singh et al. 2014), and *MHC-GAL4* (Marek et al. 2000) were employed for specified studies. *UAS-FOXO* lines 2 and 3 (*[y](http://flybase.org/reports/FBal0018607.html)w*; [*P{UAS-foxo.P}2*](http://flybase.org/reports/FBti0076467.html) and [*w^1118^*](http://flybase.org/reports/FBal0018186.html)*;* [*P{UASp-foxo.S}3*](http://flybase.org/reports/FBti0150363.html)), *Mef2-GAL4*, *Act5C-GAL4*, and *yw* flies were obtained from the Bloomington *Drosophila* Stock Center. Transgenic RNAi lines and appropriate controls were acquired from the Vienna *Drosophila* RNAi Center: *dFOXO* RNAi (#107786), CG14739 RNAi (#105594), and control (#60100). Crosses were conducted by mating virgin female *GAL4* driver flies with young UAS-transgene males. All experiments were performed on female flies collected no more than 8 hours post-eclosion.

For GeneSwitch driver experiments, flies were placed on standard media immediately after eclosion. At two days of age, they were placed on media topped with 100 μL of vehicle (100% ethanol) or with RU486 diluted to various doses in 100% ethanol. Flies were transferred each day onto new food with drug or vehicle for seven consecutive days.

*Heart tube analysis*

Semi-intact *D. melanogaster* heart tubes (Fig 1B) were prepared under oxygenated adult hemolymph (AH) at 25°C as described by (Vogler and Ocorr 2009). Cardiac performance was evaluated via high-speed video microscopy and Semi-automated Optical Heartbeat Analysis (SOHA), a free, custom-written motion analysis program (Fink et al. 2009; Vogler and Ocorr 2009; Cammarato et al. 2015; Kaushik et al. 2015). High-speed videos of beating hearts were recorded with a Hamamatsu Orca-Flash 2.8 digital camera on a Leica DM5000B DIC microscope fitted with a 10X immersion lens. Various indices of cardiac performance were calculated as previously described (Cammarato et al. 2008; Viswanathan et al. 2014; Cammarato et al. 2015; Kaushik et al. 2015). Relengthening rate was determined as the difference between systolic and diastolic diameter per the time interval between the end of isometric contraction and the beginning of isovolumic relaxation (Kaushik et al. 2011; Kaushik et al. 2015).

The myogenic “cardiac output” of the *D. melanogaster* heart tube was calculated as follows. Individual frames of beating hearts during peak diastole and peak systole were obtained from high-speed movies taken at various ages and genotypes as described. A two-dimensional area was determined for a specific length (*L*), which generally encompassed abdominal segments two through four of the heart tubes (Fig 1B) for systole and diastole, and from this an average diameter for the segment of each heart was ascertained. Since, geometrically, this segment of the *D. melanogaster* heart tube is relatively uniform, it was modeled as a cylinder. $\pi r^{2}\cdot L$ was used to determine average systolic and diastolic volumes over the designated length for each heart to provide a “stroke volume” (diastolic – systolic volume). Cardiac output (nL/sec) was calculated as stroke volume ∙ heart rate. Significance was determined as below.

Atomic Force Microscopy (AFM)-based nanoindentation to determine transverse stiffness of the conical chamber (Fig 1B) was performed with an Asylum Research MFP-3D Bio Atomic Force Microscope mounted on a Nikon Ti-U fluorescence inverted microscope with a 120 pN/nm silicon nitride cantilever premounted with a 2-µm radius borosilicate sphere (Novascan Technologies, Ames, IA) as described previously (Kaushik et al. 2011; Viswanathan et al. 2014; Kaushik et al. 2015). Beating *D. melanogaster* hearts were dissected under oxygenated AH at 25°C (Vogler and Ocorr 2009) (Fig 1B), immobilized on glass coverslips, and arrested by administration of 10 mM EGTA in AH. Eight force curves were obtained for each conical chamber from discrete locations at the ventral midline from one- and five-week control (*GMH5-GAL4 x* *yw*, n = 25, 31) and *dFOXO*-overexpressing (*GMH5-GAL4 > UAS-FOXO*, n = 25, 24) flies. The five-week time point was chosen since the procedure for exposing the conical chamber for AFM is delicate, and as flies age beyond five weeks, the risk of tissue injury during dissection is elevated. After indentation, hearts were washed with fresh AH to restore myogenic contraction. Force indentation curves were analyzed with custom-written software in MATLAB to calculate stiffness or myocardial elastic modulus (*E*, in kPa). Force curves were averaged per fly. Significance was determined as below.

*Quantitation of protein content*

To quantify dFOXO, whole *D. melanogaster* thoraces (two per biological replicate) were lysed in Laemmli sample buffer and subjected to SDS-PAGE, transferred to nitrocellulose, blocked in LiCor blocking buffer, and probed with rabbit anti-dFOXO (Slaidina et al. 2009) and goat anti-GAPDH (Invitrogen) antibodies. After incubation in IRDye secondary antibodies (LI-COR Biosciences), membranes were scanned using the Odyssey Infrared imager (LI-COR Biosciences, λ = 700 and 800 nm) and analyzed via Odyssey Application software (v3.030, LI-COR Biosciences). Mean intensity values for dFOXO from 8 - 16 biological replicates with two – three technical replicates each were normalized to GAPDH signals. Technical replicates were defined as multiple lanes containing the same biological replicate, and biological replicates were defined as different biological samples. Significant differences were assessed as below.

Ubiquitinated protein content was quantified as described above, from 6 – 10 isolated heart tubes. The seven-week time point was chosen here because although five and seven weeks of age are both considered “old” in *D. melanogaster*, gene expression changes (determined from microarray analysis; see below and Fig S5) typically precede phenotypic changes. Membranes were blocked and probed using primary mouse anti-ubiquitin (Cell Signaling) and goat anti-GAPDH (Genscript) antibodies, then incubated with IRDye secondary antibodies, scanned, and analyzed. Mean intensity values for ubiquitin from three – nine biological samples with at least two technical replicates each were normalized to GAPDH signals. Significant differences were determined as below.

*Fluorescence RNA in situ hybridization*

For quantification of *dFOXO* transcripts, hearts were dissected and exposed under oxygenated AH at 25°C (Vogler and Ocorr 2009) (Fig 1B), and contractions were arrested using 10 mM EGTA. Samples were fixed in 4% paraformaldehyde for 30 minutes, washed three times in PBST, and stored in a 96-well plate in PBS at 4°C overnight. *In situ* hybridization was performed as previously reported (Viswanathan et al. 2016) using the QuantiGene ® ViewRNA ™ Cell Assay kit from Panomics according to the manufacturer’s suggested protocol (Taylor et al. 2009), modified for use in a 96-well plate, with sequence specific probes designed to detect *dFOXO* and *GAPDH* mRNA (catalog numbers VF1-18189 and VF6-18191, respectively). Hearts were visualized by confocal microscopy with a Leica TCS SPE RGBV confocal microscope at 40X. Care was taken not to record signal from messages in the non-cardiac, ventral muscle layer that covers the dorsal vessel.

For quantification of transcripts directly from confocal micrographs, channels were separated and colors converted to grayscale. Images were opened in ImageJ, changed to 8-bit, and the threshold adjusted using the program’s default modified IsoData algorithm to an upper limit of 150 and a lower limit of 55 based arbitrarily on the first set of hearts analyzed and maintained throughout analysis. Regions of interest, which included only cardiomyocytes, were outlined using the freehand selection tool. The number of particles, corresponding to *dFOXO* and *GAPDH* messages, was determined. *dFOXO* particle number was normalized to *GAPDH* particle number for cardiomyocytes from each of 16 – 20 hearts per genotype. Significance was assessed as below.

*Microarray analysis*

Roughly 30 hearts from each genotype/ age were dissected under oxygenated AH at 25°C (Vogler and Ocorr 2009), removed, and homogenized directly in TRIzol (Invitrogen). RNA was purified (Qiagen), reverse transcribed into cDNA, and amplified via the WT-ovation Pico RNA Amplification system (NuGEN), Agencourt RNAClean (Beckman Coulter), and DNA Clean & Concentrator-25s columns (Zymo Research). cDNA was fragmented and biotin labeled for analysis on Affymetrix GeneChip arrays. Two biological replicate hybridizations of the labeled cardiac cDNA to the Affymetrix GeneChip arrays and expression analyses were performed at the UCSD/ Veterans Medical Research Foundation GeneChip Microarray Core facility. To determine differentially expressed genes, by age and genotype, ANOVA interaction *p*-values were computed using LIMMA (Wettenhall and Smyth 2004). Raw Affymetrix microarray data has been uploaded to GEO and can be accessed with the following access number: GSE73205. We identified 2,315 genes to be differentially expressed (interaction *p* <0.05, based on a moderated T statistic which is equivalent to FDR adjusted p<0.2, and |fold change| > 2 in at least one of all possible pairwise comparisons from each of the four groups) between control and *dFOXO*-overexpressing flies at one and five weeks of age. Genes were clustered with AltAnalyze (Emig et al. 2010) and clusters were then analyzed for enrichment of functional terms (i.e. Gene Ontology, Kyoto Encyclopedia of Genes and Genomes (KEGG) pathways) using GO-Elite (Zambon et al. 2012). Significance was determined as below using one-week control as a baseline.

Enrichment analysis of insect and vertebrate forkhead DNA binding sites was conducted with oPOSSUM (Kwon et al. 2012). Genes encoding mRNAs significantly upregulated in five-week *GMH5-GAL4* > *UAS-dFOXO* vs. five-week *GMH5-GAL4* x *yw* control hearts (1,324 genes, Fold > 2, *p* < 0.05) were submitted for enrichment analysis of evolutionarily conserved forkhead binding sites within 200bp up- and down-stream of the transcriptional start site (TSS). The 200bp window resulted in the highest level of forkhead family sites compared with 2000 and 500 bp query windows surrounding the TSS. This is in concordance with variations of FOXO3 binding site enrichment surrounding the TSS site identified by ChIP-seq (Eijkelenboom et al. 2013). DNA binding sites were queried using matrices from the JASPAR database (Bryne et al. 2008).

*Imaging of D. melanogaster cardiomyocyte nuclei and larvae*

Hearts of two-day flies expressing *UAS-stinger*, a nuclear GFP-harboring transgenic line, were dissected under oxygenated AH at 25°C (Vogler and Ocorr 2009) (Fig 1B). Contractions were arrested by exposure to 10 mM EGTA. Samples were fixed (4% paraformaldehyde, 1X PBS), rinsed, mounted on glass slides in ProLong® Gold antifade reagent with DAPI (Life Technologies), and viewed with a Leica TCS SPE RGBV confocal microscope fitted with a 40X oil immersion lens (Alayari et al. 2009; Viswanathan et al. 2014; Kaushik et al. 2015). Nuclear fluorescence intensity was quantified using Leica LAS AF software. All hearts per experiment were imaged once over the period of one day to avoid differences in laser intensity and photobleaching. Significant differences were calculated as below.

To visualize dFOXO protein localization in the *D. melanogaster* myocardium, hearts were dissected and exposed under oxygenated AH at 25°C, and contractions were arrested using 10 mM EGTA. Samples were fixed (4% paraformaldehyde, 1X PBS), washed three times in PBS, and a final time in PBST. Samples were transferred to a 96-well plate and incubated at 4°C overnight in 1:1000 primary rabbit anti-dFOXO (Slaidina et al. 2009). They were washed and incubated in 1:1000 secondary CY5 goat anti-rabbit (Life Technologies), washed, and mounted on glass slides in ProLong® Gold antifade reagent with DAPI (Life Technologies). Hearts were imaged as above.

*Hand^4.2^-GAL4* was crossed with *UAS-dFOXO* line 1 or *yw* control. At 4 days post-fertilization, larvae from both crosses were imaged with a Leica M165FC microscope fitted with a Leica EC3 camera.

*Statistical Analyses*

Appropriate sample size was determined based on previous studies (Kaushik et al. 2015). All statistics and analyses were performed using GraphPad Prism 5.01. Values were determined to be normally distributed by post-hoc normality tests. Significant differences between genotype and age, and interaction effects, were determined using two-way analysis of variance (ANOVA) with Bonferroni post-hoc tests. In experiments in which different ages were not investigated, one-way ANOVA or Student’s t-tests were used to determine significance. Significance was reported at *p* < 0.05. Pooled data are represented as mean ± SEM unless otherwise specified. All experiments were performed using indicated sample sizes.

**Supplemental Figures**


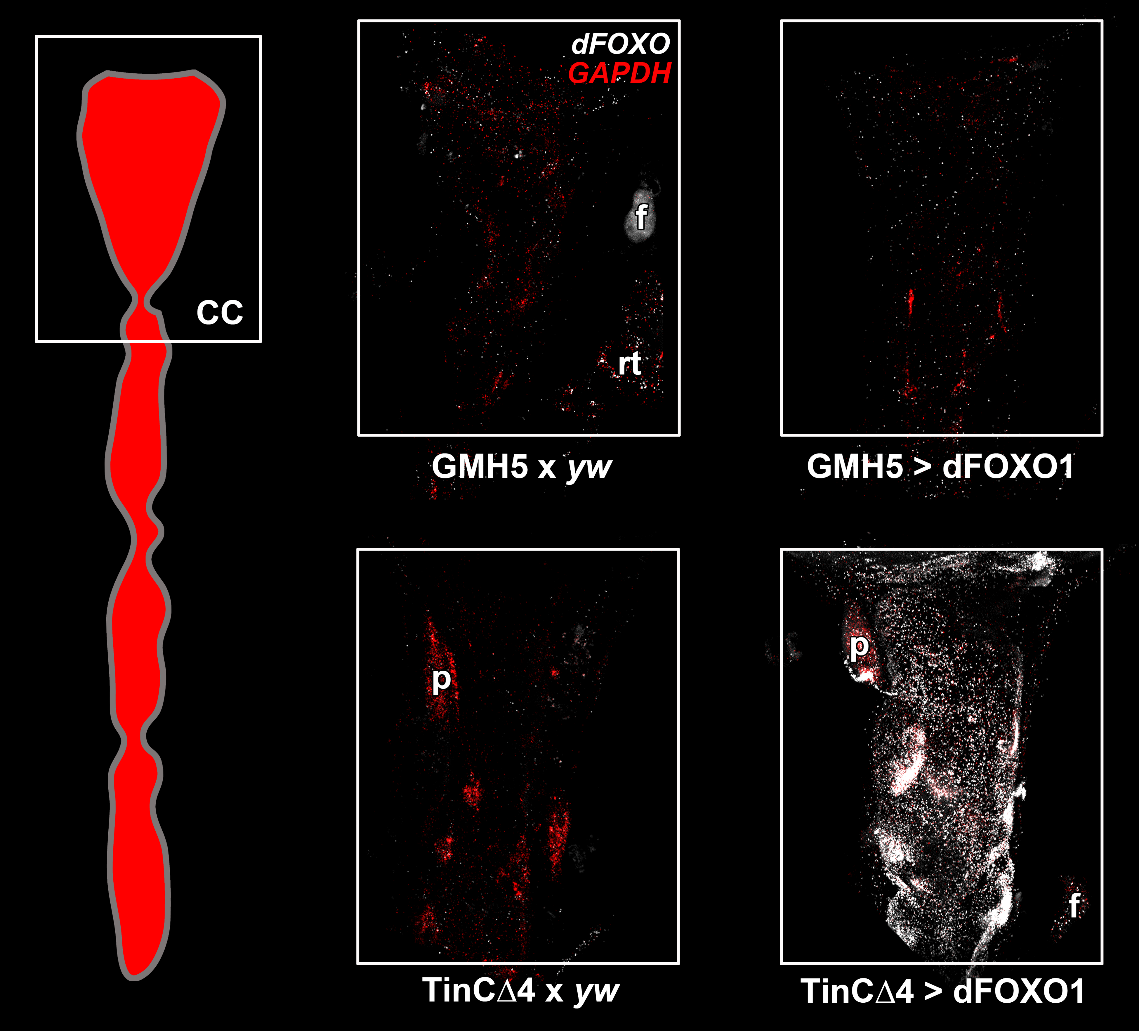


**Fig S1. *dFOXO* transcripts are more abundant in hearts overexpressing transgenic *dFOXO* compared to control.**

Images of the conical chamber (CC) of *D. melanogaster* hearts probed for *dFOXO* (white) and *GAPDH* (red) mRNA using the ViewRNA FISH cell assay. *GMH5-GAL4 > UAS-dFOXO* line 1 contained more *dFOXO/GAPDH* particles than control *GMH5 x yw* (Fig 1). TinCΔ4-GAL4 (moderate-strength driver, see Fig 4) drove *UAS*-*dFOXO* overexpression to a much greater extent than GMH5-GAL4. Pericardial cells (p), fat (f), and body wall muscles (rt) are shown. The ViewRNA FISH method allows for the exclusion of these cells for detection and quantification of gene expression only in cardiomyocytes.


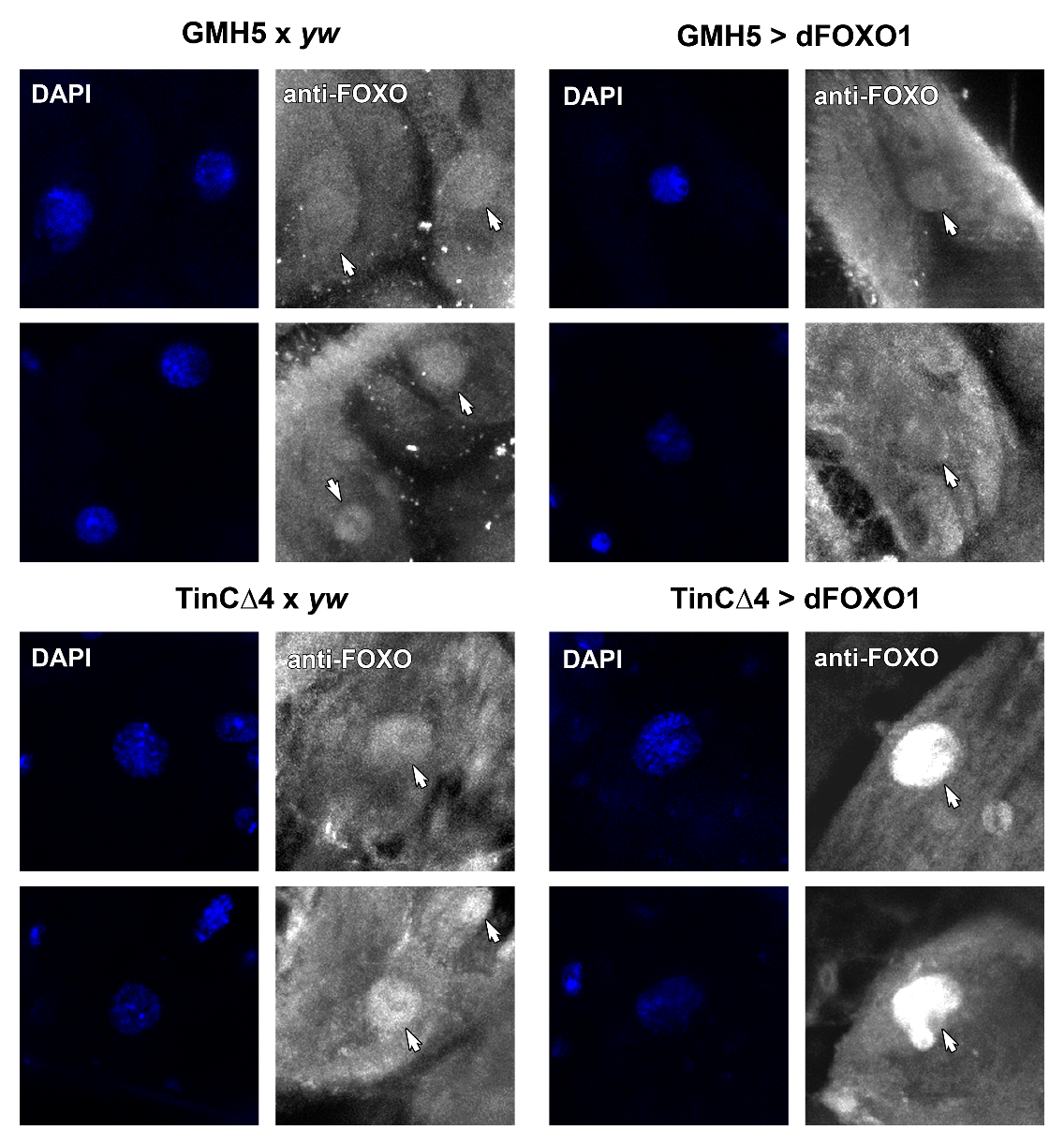


**Fig S2. Immunolocalization of dFOXO following overexpression via GMH5- or TinCΔ4-GAL4.**

Anti-dFOXO and fluorescently labeled secondary antibodies were used to determine the extent of dFOXO nuclear accumulation following overexpression in five-day female *D. melanogaster* hearts. Our immunostaining approach could not definitively resolve elevated dFOXO in cardiomyocyte nuclei (white arrows), above background, when weakly overexpressed via the GMH5-GAL4 driver. However, when *dFOXO* was overexpressed using the strong TinCΔ4-GAL4 driver, robust nuclear fluorescence was emitted, clearly indicating nuclear accumulation vs. control.





**Fig S3**. **GMH5-GAL4-driven *dFOXO* overexpression suppresses heart function decline in aging *D. melanogaster*.**

High-speed video microscopy and motion analysis software were used to analyze beating *D. melanogaster* hearts at one, five, and seven weeks of age. *GMH5-GAL4 > UAS-dFOXO* line 1 exhibited significant improvement in cardiac output, heart period, arrhythmicity index, diastolic interval, and relengthening rate compared to control hearts with age (n = 50, two-way ANOVA, Bonferroni post-hoc test). These data illustrate the progressive nature of cardiac decline and the beneficial effect of *dFOXO* overexpression over time.





**Fig S4**. **Amelioration of functional decline in aging *dFOXO*-overexpressing hearts is corroborated with a second *UAS-dFOXO* transgenic line.**

High-speed video microscopy and motion analysis software were used to analyze beating *D. melanogaster* hearts at one and seven weeks of age. No significant differences were observed between the two groups (*GMH5-GAL4* > *UAS-dFOXO* line 1 vs. *GMH5-GAL4* > *UAS-dFOXO* line 2) in cardiac output, heart period, arrhythmicity index, diastolic interval, and myocardial relengthening rate (n = 20-52, two-way ANOVA). Significant differences were seen between both *dFOXO*-overexpressing hearts and controls.

**
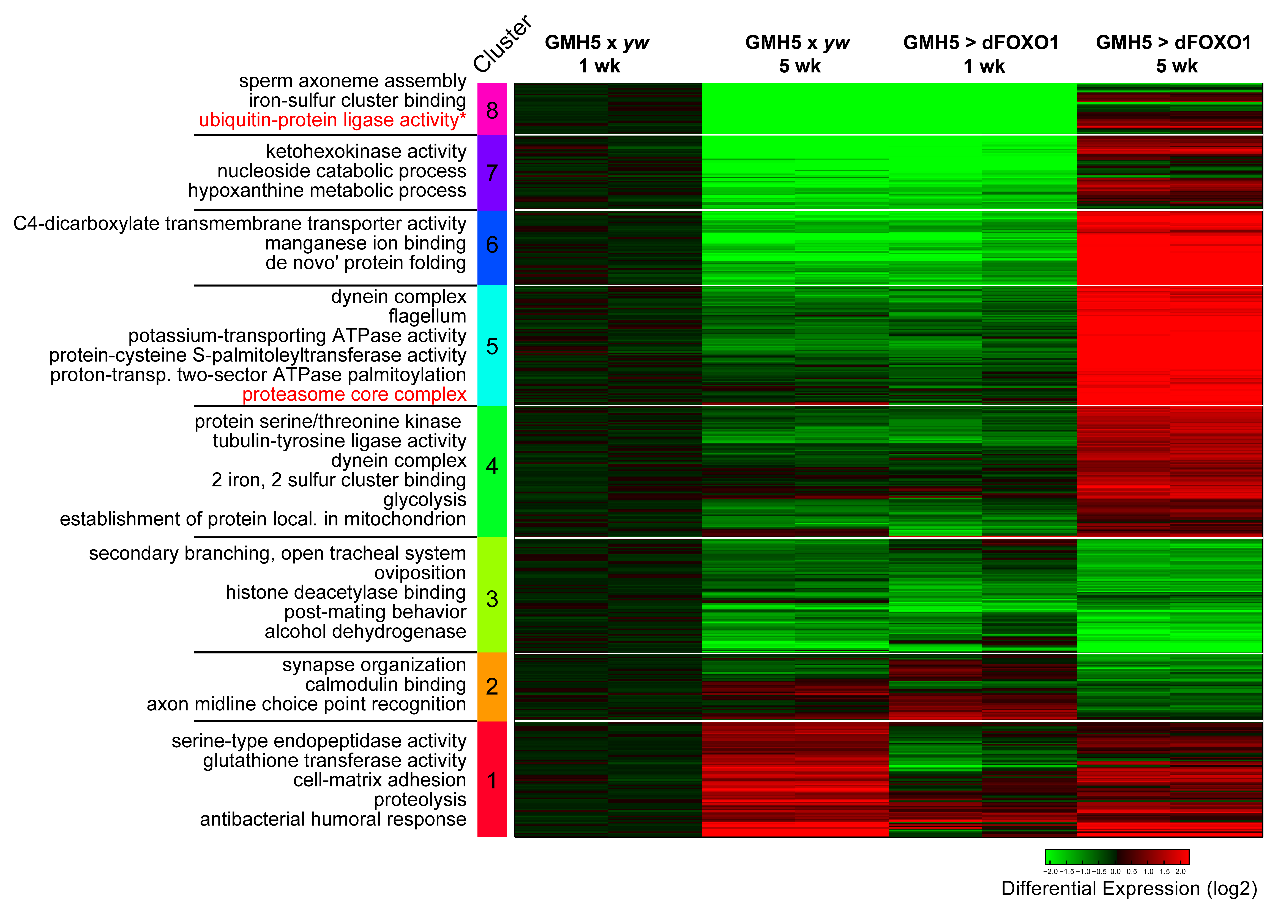
**

**Fig S5**. **Cardiac-specific *dFOXO* overexpression via GMH5-GAL4 influences the transcription of genes associated with many biological functions and components including the UPS**.

Heat map displaying changes (green indicates decrease, red indicates increase) in gene transcripts (2,315 mRNAs, see detailed Experimental Procedures online) in hearts of flies of different ages and genotypes compared to one-week control hearts (black column), as resolved by microarray analysis. Genes were clustered by pattern of global transcript changes. UPS-associated genes are contained within clusters 5 and 8. Note that the UPS-associated gene expression profile in young *dFOXO*-overexpressing hearts resembled that of aged control hearts (clusters 5 and 8), but this did not negatively impact cardiac function. It is possible that autophagy compensates for any inadequacies of the UPS in young *dFOXO*-overexpressing hearts, as UPS and autophagy have been increasingly linked in cardiomyocytes (Wang and Wang 2015), yet this compensation does not occur in aged control hearts. As aging progressed, a panoply of genes associated with the UPS vs. autophagy was upregulated in *dFOXO*-overexpressing hearts and likely contributed to preservation of cardiac function.


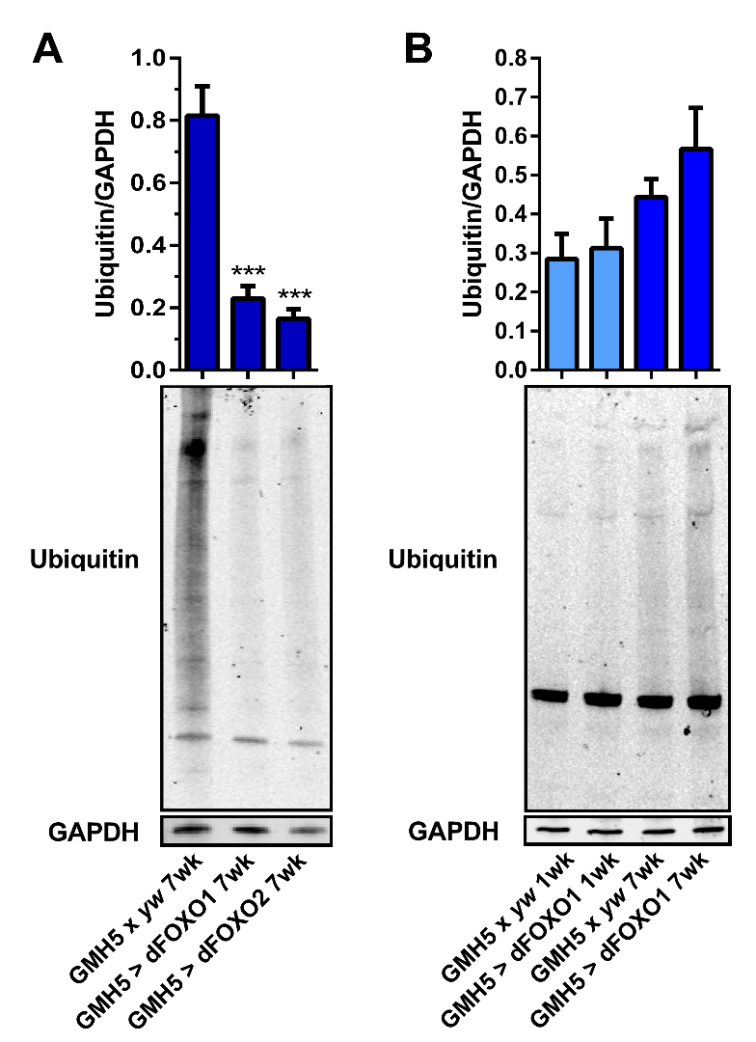


**Fig S6**. **GMH5-GAL4-driven *dFOXO* overexpression in lines 1 and 2 reduces ubiquitinated protein in aged fly hearts, while ubiquitin content in aged IFMs is not affected.**

A. Quantitative western blot analysis was performed on seven-week hearts for ubiquitin and GAPDH (endogenous control). Cardiac-specific overexpression of *dFOXO* using *UAS-dFOXO* line 2 significantly reduced the ubiquitinated protein content in aged hearts compared to controls (0.17 ± 0.03 vs. 0.82 + 0.10), corroborating the results from *UAS-dFOXO* line 1 (n = 6, ****p* < 0.001, one-way ANOVA). B. Dissected IFMs from one- and seven-week flies were also probed with anti-ubiquitin and anti-GAPDH antibodies. Overexpression of *dFOXO* in cardiomyocytes does not reduce ubiquitinated protein content in their IFMs compared with that in aged-matched controls (one-week: 0.31 ± 0.07 vs. 0.28 ± 0.06; seven-week: 0.57 ± 0.10 vs. 0.41 ± 0.07; n = 5) suggesting beneficial responses are confined to the heart.





**Fig S7**. **Sequence alignments of UPS-associated proteins.**

Sequence alignment, generated by the DRSC Integrative Ortholog Prediction Tool (DIOPT) (http://www.flyrnai.org/cgi-bin/DRSC_orthologs.pl), of proteins involved in the UPS from *D. melanogaster* and *Homo sapiens* reveals high conservation (Hu et al. 2011). UBE2H (CG14739) and UBE2N (CG3473) are E2 ubiquitin conjugating enzymes, and RNF41 (CG9014) is an E3 ubiquitin ligase. An (*) indicates identical residues, a (:) indicates amino acids with high structural similarity, and a (.) indicates amino acids with low structural similarity.


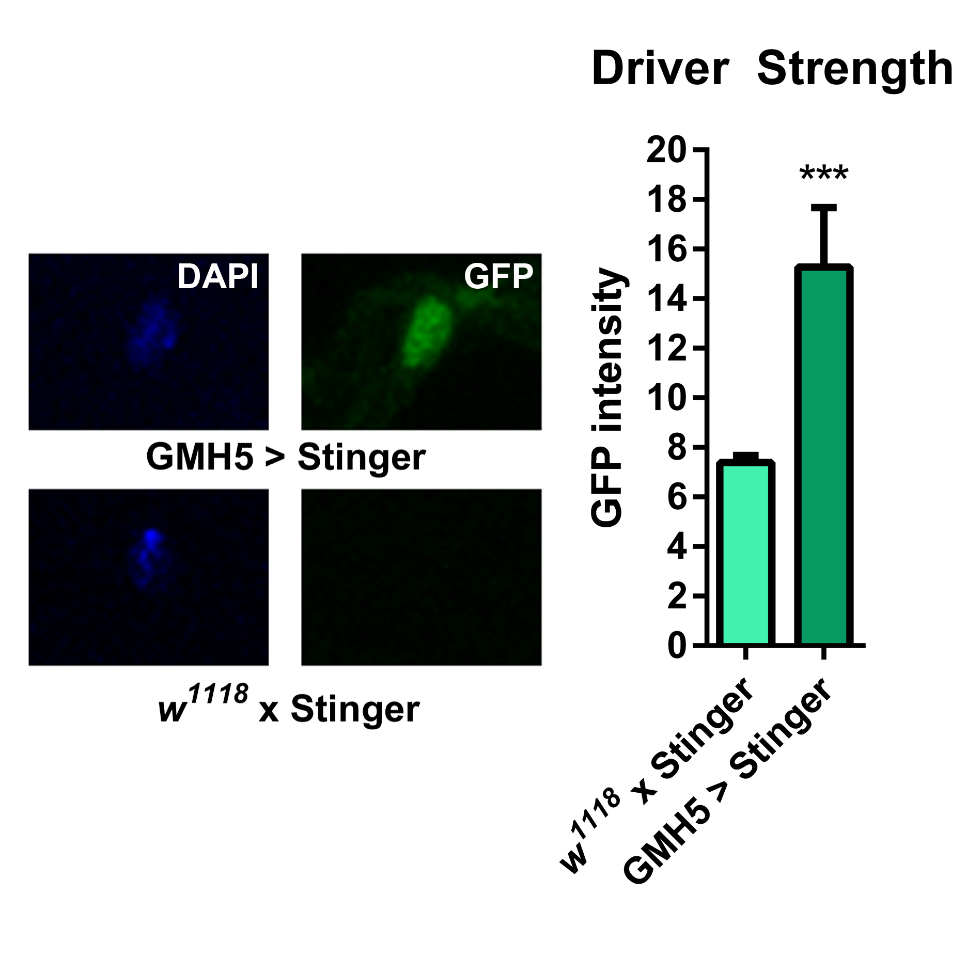


**Fig S8.** **The GMH5-GAL4 driver overexpresses UAS-controlled transgenes in cardiomyocytes above endogenous levels.**

At high excitation intensity (λ = 488nm, 20.64%), fluorescence emission intensity produced by *GMH5-GAL4 > UAS-Stinger* cardiomyocyte nuclei was significantly greater than control (15 ± 2.4 A.U. vs. 7.4 ± 0.3 A.U.; n > 100 nuclei, ****p* < 0.001, Student’s t-test).


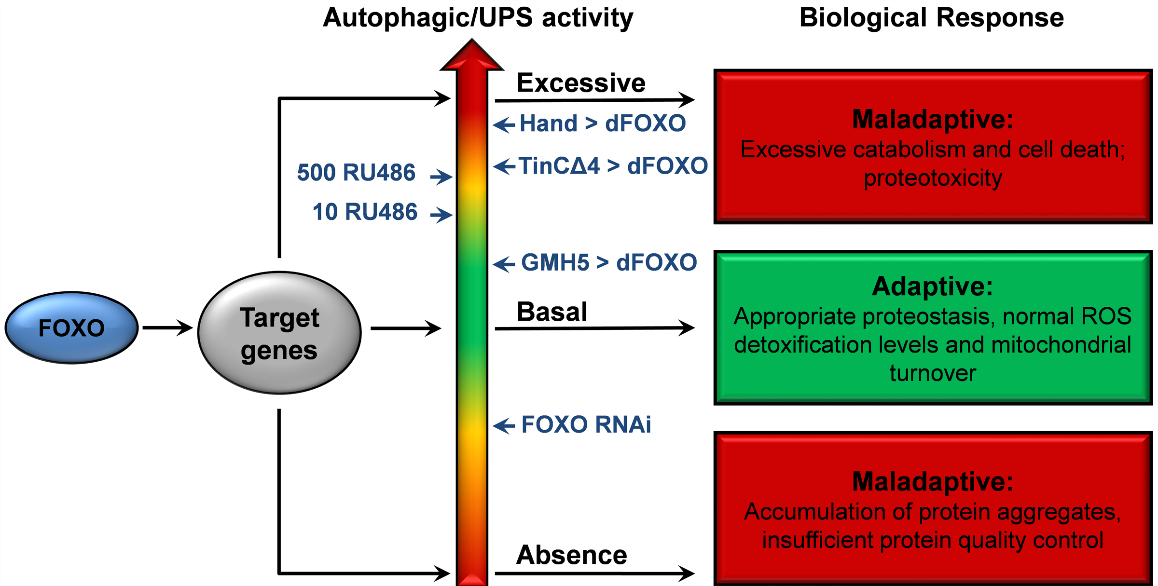


**Fig S9**. **Discrete quantities of dFOXO potentially result in graded levels of PQC activity that can positively or negatively affect the heart.**

FOXO regulates the transcription of genes associated with autophagy and the UPS. Distinct amounts of dFOXO induced an array of myocardial responses in flies consistent with the transcription factor promoting graded levels of autophagic and UPS activity as previously suggested (Rothermel and Hill 2008; Ferdous et al. 2010). Maintaining *dFOXO* at slightly elevated expression levels improved cardiac performance during aging, conceivably due to enhanced UPS activity. Maladaptive responses observed in *D. melanogaster* hearts with excessive *dFOXO* overexpression or suppression may be due in part to rampant autophagy or insufficient PQC activity, respectively. Blue arrows indicate where on the continuum each dose of *dFOXO* overexpression is predicted to lie based on cardiac responses after using drivers of differing strengths, RU486-induced expression, and RNAi-mediated *dFOXO* knockdown.

**Supplemental Tables**

| **UPS Function** | **Gene** | **Control fold change** | ***GMH5 > FOXO* fold change** | **Interaction**  ***p*-value** | **FDR-adjusted *p*-value** |
| --- | --- | --- | --- | --- | --- |
| 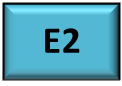 | ***CG14739*** | **-18.9** | **13.9** | **< 0.001** | **< 0.001** |
|  | ***CG10862*** | **-3.4** | **18.7** | **< 0.001** | **< 0.001** |
|  | ***CG17030*** | **-6.1** | **10.6** | **< 0.001** | **< 0.001** |
|  | ***CG3473*** | **-1.7** | **9.3** | **< 0.001** | **< 0.001** |
|  | ***CG9602*** | **-15.7** | **8.7** | **< 0.01** | **< 0.001** |
|  | ***CG2574*** | **-4.3** | **5.0** | **< 0.01** | **< 0.001** |
|  | ***Ubc84D*** | **-1.7** | **4.8** | **< 0.01** | **< 0.01** |
|  | ***CG5440*** | **1.0** | **3.1** | **< 0.001** | **< 0.01** |
| 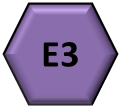 | ***Sinah*** | **-13.7** | **16.0** | **< 0.001** | **< 0.001** |
|  | ***CG9014*** | **-4.0** | **9.4** | **< 0.01** | **< 0.001** |
|  | ***Roc1b*** | **-1.3** | **1.5** | **< 0.05** | **NS** |
|  | ***CG5087*** | **-1.1** | **1.3** | **< 0.05** | **NS** |
| Proteasome subunit alpha | ***Prosalpha3T*** | **-1.6** | **17.8** | **< 0.001** | **< 0.001** |
| De-ubiquitinases | ***Uch-L3/L5*** | **-25.6** | **19.0** | **< 0.001** | **< 0.001** |
|  | ***CG14619*** | **-1.0** | **8.1** | **< 0.001** | **< 0.001** |

**Table S1. UPS-associated genes whose transcription is significantly altered in hearts with modest *dFOXO* overexpression compared to controls.**

**
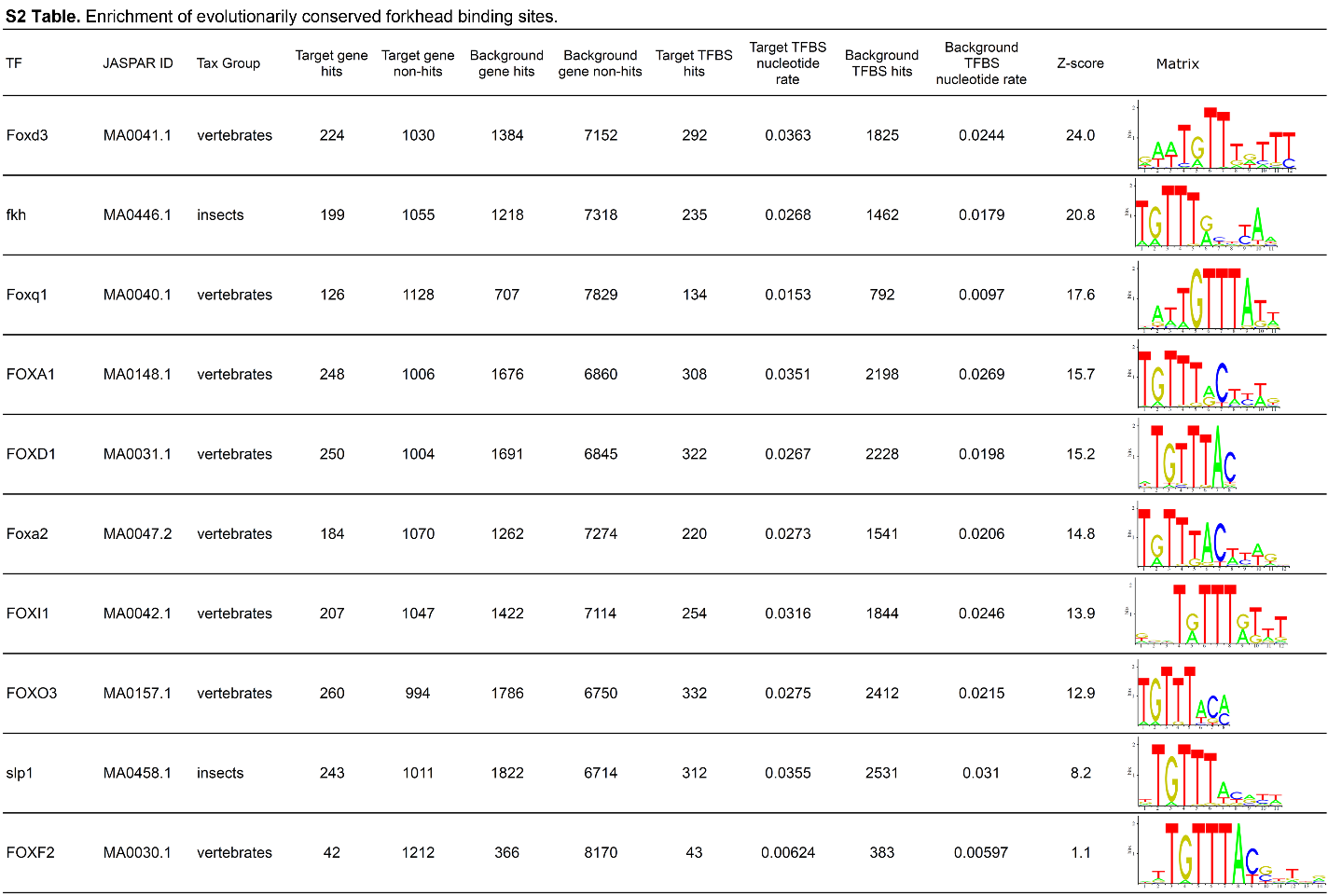
**

**Table S2. Enrichment of evolutionarily conserved forkhead binding sites.**

To confirm *dFOXO* overexpression increased the amount of active dFOXO in cardiomyocyte nuclei, a bioinformatics approach was employed and demonstrated that expression of forkhead family gene targets was altered in the microarray data set (GEO access number: GSE73205). We hypothesized that the proximal promoters of genes upregulated in five-week *GMH5-GAL4 > UAS-dFOXO* line 1 hearts compared to five-week controls (Fold>2, *p*<.05) would contain an enrichment of evolutionarily conserved forkhead DNA binding sites near the transcriptional start site (within 200 bp) compared to a background set of unaltered genes measured on the microarrays. Using oPOSSUM (Kwon et al. 2012) we found a highly significant enrichment of all but one conserved forkhead binding sites queried from the JASPAR database (Bryne et al. 2008). All matrices contained a core forkhead sequence of TG/ATTT. Ranking enriched forkhead sites by significance score revealed the insect sites (fkh) as the second most enriched, below vertebrate FoxD3 sites, with 2.6% of upregulated genes containing the site compared to only 1.8% of background non-regulated genes (Z score=20.8; *p*<1e^-5^). Thus, transgenic dFOXO appeared to actively target and regulate gene expression in cardiomyocytes.

| **Ontology name** | **Gene** | **Human homolog** | **Control fold change** | **GMH5 > FOXO fold change** | **FDR-adjusted p-value** | **References** |
| --- | --- | --- | --- | --- | --- | --- |
| Structural | Cg25C | COL4A1 (Collagen type IV alpha 1) | -1.12 | 1.1 | <0.05 | (Hollfelder et al. 2014) |
|  | Rtnl2 (tm-like) | RTN1/2/3 (reticulon 1/2/3) | -1.4 | 8.15 | <0.05 | (Tarasov et al. 2009) |
|  | Actn | ACTN2 (Actinin alpha 2) | -1.6 | -1.36 | <0.05 | (Taghli-Lamallem et al. 2014)  (Vojta et al. 1996) |
| Glycolysis | CG12229 | PKM (pyruvate kinase) | -7.47 | 12.65 | <0.05 | (Curtis et al. 2007) |
|  | CG2964 | PKLR (pyruvate kinase) | -2.32 | 2.48 | <0.05 | (Li et al. 2016) |
|  | CG33791 | OGDH (oxoglutarate dehydrogenase) | -8.99 | 6.26 | <0.05 | (Durham et al. 2014) |
|  | CG5432 | ALDOA (aldose, fructose-biphosphate A) | -3.33 | 3.27 | <0.05 | (Quinlan et al. 2013) |
|  | CG9961 | PGK2/1 (phosphoglycerate kinase 2/1) | -1.16 | 2.56 | <0.05 | (Curtis et al. 2007)  (Moriyama et al. 2014) |
|  | Hex-t2 | GCK (glucokinase)  HK1/3 (hexokinase 1/3) | -10.23 | 8.07 | <0.01 | (Arking et al. 2000)  (Xu et al. 2014) |
|  | Pglym87 (CG17645) | PGAM2 (phosphoglycerate mutase 2) | -5.55 | 4.08 | <0.05 | (Neely et al. 2010) |
| Nucleoside catabolic process/Growth factor activity | Adgf-B  Adgf-E | CECR1 (adenosine deaminase-related growth factor B/E) | -4.76  -3.84 | 2.32  3.41 | <0.05  <0.01 |  |
|  | CG8349 | MAGED4B (melanoma antigen family) | -3.13 | 6.99 | <0.05 |  |
|  | Msi (Musashi) | CECR1 | -4.71 | 5.73 | <0.05 | (Bai et al. 2013) |
|  | Daw | TGFB1 (transforming growth factor beta | -1.36 | 2.05 | <0.05 | (Toba et al. 2016) |
| Acid-amino acid ligase activity | CG16716 | TTLL6 (tubulin tyrosine ligase-like 6) | -6.88 | 16.73 | <0.05 | (Neely et al. 2010) |
|  | CG16894 | AKTIP (AKT interacting protein) | -4.48 | 16.68 | <0.01 |  |
|  | CG31773 | FPGS (folylpoly-glutamate synthase) | -3.36 | 6.01 | <0.05 |  |
|  | skpF | SKP1 (S-phase kinase-associated protein 1) | -4.59 | 7.89 | <0.05 | (Wagner et al. 2001) |
| Mitochondrial matrix | CG10749 | STRBP (spermatid perinuclear RNA binding protein) previously ILF3L | -14.27 | 7.92 | <0.05 |  |
|  | CG14740 | CS (citrate synthase) | -18.73 | 12.74 | <0.05 | (Molina et al. 2016) |
|  | CG3483 | IDH3A (isocitrate dehydrogenase 3 (NAD+) alpha | -4.37 | 3.43 | <0.05 | (Fiorini et al. 2013) |
|  | CG4434 | GLUD1 (glutamate dehydrogenase 1) | -13.83 | 16.85 | <0.01 | (Neely et al. 2010) |
|  | P5CDh2 (CG33092) | ALDH4A1 (aldehyde dehydrogenase) | -5.68 | 5.68 | <0.05 |  |
| **Ontology name** | **Gene** | **Human homolog** | **Control fold change** | **GMH5 > FOXO fold change** | **FDR-adjusted p-value** | **References** |
| Mitochondrial inner membrane | CG12201 | SLC25A22 (mitochondrial carrier: glutamate) | -9.89 | 6.96 | <0.05 |  |
|  | CG14077 | COX6A1 (cytochrome c oxidase) | -8.70  -5.02 | 5.05  8.72 | <0.01  <0.01 | (Boczonadi et al. 2015) |
|  | CG1724 | TIMM17B (translocase of inner mitochondrial membrane 17) | -1.86 | 2.69 | <0.05 | (Neely et al. 2010) |
|  | CG5265 | CRAT (carnitine O-acetyltransferase) | -7.59 | 8.65 | <0.05 | (Noland et al. 2009) |
|  | CG6914 | NDUFA7 (NADH:ubiquinone oxidoreductase) | -2.72 | 6.87 | <0.05 |  |
|  | CG7514 | UCP1 (uncoupling protein 1) | -3.98 | 6.02 | <0.05 | (Neely et al. 2010) |
|  | Cyt-c-d | CYCS (cytochrome c, somatic) | -3.88 | 9.24 | <0.05 |  |
|  | Tim13 | TIMM13 (translocase of inner mitochondrial membrane 17) | -4.37 | 9.29 | <0.01 |  |
|  | ttm2  ttm3 | TIMM50 | -7.59  -4.42 | 13.64  9.92 | <0.01  <0.01 |  |
| Oxidation-reduction process | CG13611 (FAD/NAD binding) | None identified | -4.095 | 3.87 | <0.05 |  |
|  | CG18193 (COX7AL) | COX7A1 (cytochrome c oxidase subunit 7A1) | -4.04 | 12.50 | <0.01 | (Ronn et al. 2008) |
|  | CG2336 (aldehyde de-hydrogenase) | None identified | -5.12 | 13.01 | <0.05 |  |
|  | CG31546 | HSD17B14 (hydroxy-steroid (17-beta) dehydrogenase 14) | -3.83 | 3.04 | <0.05 |  |
|  | CG32655 | None identified | -11.47 | 11.74 | <0.01 |  |
|  | CG33791 | None identified | -9.00 | 6.26 | <0.05 |  |
|  | CG4836 | SORD (sorbitol dehydrogenase) | -32.17 | 18.11 | <0.05 | (Curtis et al. 2007) |
|  | CG6914 | NDUFA7 (NADH:ubiquinone oxidoreductase) | -2.72 | 6.87 | <0.05 | (Ananthakrishnan et al. 2011) |
|  | CG7140 | G6PD (glucose-6-phosphate dehydrogenase) | -4.22 | 5.31 | <0.05 |  |
|  | CG7311 | GPD2 (glycerol-3-phosphate dehydrogenase 2) | -16.00 | 7.90 | <0.05 |  |
|  | Fer3HCH | FTMT/FTH1 (Ferritin, mitochondrial/heavy polypeptide 1) | -2.67 | 3.37 | <0.05 | (den Hoed et al. 2013) |
|  | Pxd | PXDN (peroxidasin) | -2.54 | 2.45 | <0.05 |  |
|  | TrxT (thio-redoxin) | TXNDC2 (thioredoxin domain) | -14.43 | 17.60 | <0.01 | (Neely et al. 2010) |
|  | mex1 | AP3B1 (adaptor related protein complex) | -1.38 | 2.53 | <0.01 |  |
| **Ontology name** | **Gene** | **Human homolog** | **Control fold change** | **GMH5 > FOXO fold change** | **FDR-adjusted p-value** | **References** |
| NAD binding (oxidation-reduction process; isocitrate dehydrogenase activity) | CG32026 | IDH3A (isocitrate dehydrogenase 3 alpha) | -4.02 | 7.04 | <0.05 | (Neely et al. 2010) |
|  | CG43343 (CG31169) | GPD1L (glycerol-3-phosphate dehydrogenase 1-like) | -2.94 | 1.99 | <0.05 |  |
|  | Menl-2 | ME1/2/3 (Malic enzyme 1/2/3) | -11.90 | 10.42 | <0.05 |  |
| Oxygen transport | glob2  glob3 | Globin-like; protoglobin | -2.77  -1.36 | 10.78  5.01 | <0.05  <0.05 |  |

**Table S3. Additional pathways and genes that are differentially regulated with age between *GMH5-GAL4* x *yw* vs. *dFOXO*-overexpressing hearts that may contribute to dFOXO-directed improvements in heart function during non-pathological aging.**

Genes listed represent those whose transcription was significantly downregulated in *GMH5-GAL4* x *yw* control hearts with age and upregulated in *GMH5-GAL4 > UAS-dFOXO* line 1 hearts with age. Along with PQC, pathways noted above are remarkably important for normal cardiomyocyte function. Thus, it stands to reason that the nonpathological, age-dependent downregulation of genes involved in these pathways has a detrimental impact on cardiac performance over time. References listed include studies that describe the association of specific genes with dysfunction upon aging or stress, many specifically in myocardial tissue or muscle. Neely et al. is listed in reference to *D. melanogaster* genes that when suppressed via RNAi in myocardium induced premature death (Neely et al. 2010). Note: FDR-adjusted p-value denotes interaction effects.


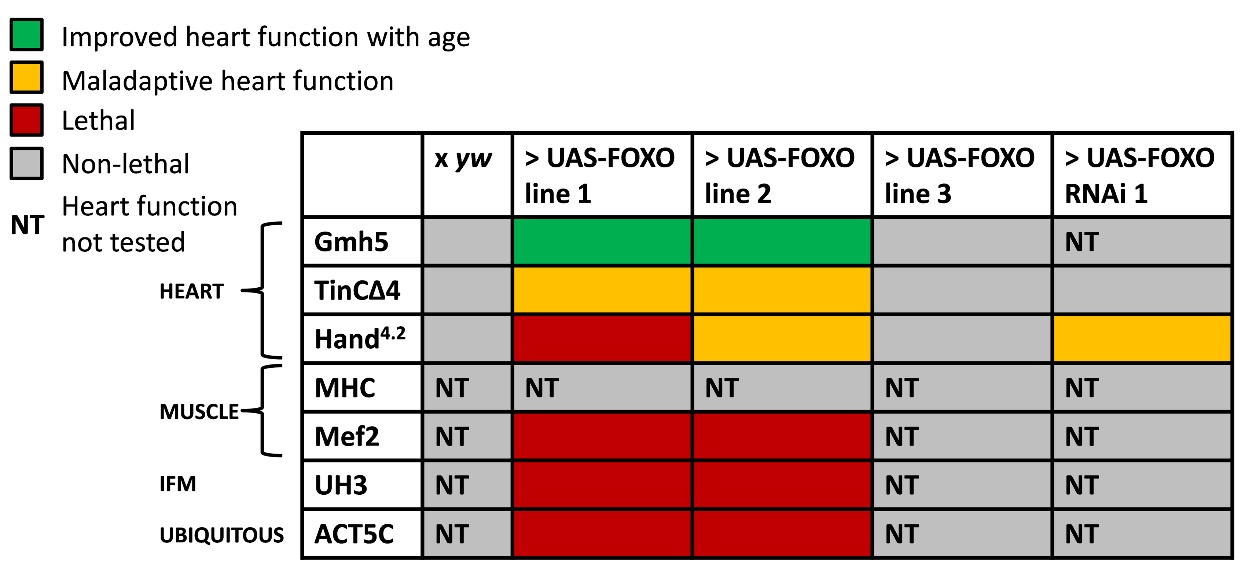


**Table S4. Cardiac and developmental consequences resulting from combinations of GAL4 drivers with various *UAS-dFOXO* overexpression and knockdown constructs.**

Green boxes indicate improved cardiac function over time, yellow boxes indicate maladaptive function and accelerated cardiac aging, red boxes indicate developmental lethality, and gray boxes indicate no apparent developmental alteration. NT indicates that cardiac function was not directly analyzed in progeny resulting from the cross. Interestingly, pupal lethality was observed in flies overexpressing *dFOXO* using Mef2-GAL4, which was shown to drive higher transgene expression than MHC-GAL4 (Viswanathan et al. 2015). Pupal lethality was also observed in flies overexpressing *dFOXO* ubiquitously (ACT5C-GAL4) and specifically in the IFM (UH3-GAL4).

**Supplemental References**

Alayari, N. N., G. Vogler, et al. (2009). "Fluorescent labeling of Drosophila heart structures." J Vis Exp(32).10.3791/1423 1423 [pii].

Ananthakrishnan, R., Q. Li, et al. (2011). "Aldose reductase pathway contributes to vulnerability of aging myocardium to ischemic injury." Exp Gerontol **46**(9): 762-7.10.1016/j.exger.2011.05.001.

Arking, R., V. Burde, et al. (2000). "Identical longevity phenotypes are characterized by different patterns of gene expression and oxidative damage." Exp Gerontol **35**(3): 353-73.

Bai, H., P. Kang, et al. (2013). "Activin signaling targeted by insulin/dFOXO regulates aging and muscle proteostasis in Drosophila." PLoS Genet **9**(11): e1003941.10.1371/journal.pgen.1003941.

Barolo, S., L. A. Carver, et al. (2000). "GFP and beta-galactosidase transformation vectors for promoter/enhancer analysis in Drosophila." Biotechniques **29**(4): 726, 728, 730, 732.

Boczonadi, V., M. Giunta, et al. (2015). "Investigating the role of the physiological isoform switch of cytochrome c oxidase subunits in reversible mitochondrial disease." Int J Biochem Cell Biol **63**: 32-40.10.1016/j.biocel.2015.01.025.

Bryne, J. C., E. Valen, et al. (2008). "JASPAR, the open access database of transcription factor-binding profiles: new content and tools in the 2008 update." Nucleic Acids Res **36**(Database issue): D102-6.gkm955 [pii] 10.1093/nar/gkm955.

Cammarato, A., C. M. Dambacher, et al. (2008). "Myosin transducer mutations differentially affect motor function, myofibril structure, and the performance of skeletal and cardiac muscles." Mol Biol Cell **19**(2): 553-62.E07-09-0890 [pii] 10.1091/mbc.E07-09-0890.

Cammarato, A., S. Ocorr, et al. (2015). "Enhanced assessment of contractile dynamics in Drosophila hearts." Biotechniques **58**(2): 77-80.10.2144/000114255 000114255 [pii].

Curtis, C., G. N. Landis, et al. (2007). "Transcriptional profiling of MnSOD-mediated lifespan extension in Drosophila reveals a species-general network of aging and metabolic genes." Genome Biol **8**(12): R262.10.1186/gb-2007-8-12-r262.

den Hoed, M., M. Eijgelsheim, et al. (2013). "Identification of heart rate-associated loci and their effects on cardiac conduction and rhythm disorders." Nat Genet **45**(6): 621-31.10.1038/ng.2610.

Durham, M. F., M. M. Magwire, et al. (2014). "Genome-wide analysis in Drosophila reveals age-specific effects of SNPs on fitness traits." Nat Commun **5**: 4338.10.1038/ncomms5338.

Eijkelenboom, A., M. Mokry, et al. (2013). "Genome-wide analysis of FOXO3 mediated transcription regulation through RNA polymerase II profiling." Mol Syst Biol **9**: 638.10.1038/msb.2012.74.

Emig, D., N. Salomonis, et al. (2010). "AltAnalyze and DomainGraph: analyzing and visualizing exon expression data." Nucleic Acids Res **38**(Web Server issue): W755-62.gkq405 [pii] 10.1093/nar/gkq405.

Ferdous, A., P. K. Battiprolu, et al. (2010). "FoxO, autophagy, and cardiac remodeling." J Cardiovasc Transl Res **3**(4): 355-64.10.1007/s12265-010-9200-z.

Fink, M., C. Callol-Massot, et al. (2009). "A new method for detection and quantification of heartbeat parameters in Drosophila, zebrafish, and embryonic mouse hearts." Biotechniques **46**(2): 101-13.000113078 [pii] 10.2144/000113078.

Fiorini, A., R. Sultana, et al. (2013). "Antisense directed against PS-1 gene decreases brain oxidative markers in aged senescence accelerated mice (SAMP8) and reverses learning and memory impairment: a proteomics study." Free Radic Biol Med **65**: 1-14.10.1016/j.freeradbiomed.2013.06.017.

Han, Z. and E. N. Olson (2005). "Hand is a direct target of Tinman and GATA factors during Drosophila cardiogenesis and hematopoiesis." Development **132**(15): 3525-36.dev.01899 [pii] 10.1242/dev.01899.

Hollfelder, D., M. Frasch, et al. (2014). "Distinct functions of the laminin beta LN domain and collagen IV during cardiac extracellular matrix formation and stabilization of alary muscle attachments revealed by EMS mutagenesis in Drosophila." BMC Dev Biol **14**: 26.10.1186/1471-213X-14-26.

Hu, Y., I. Flockhart, et al. (2011). "An integrative approach to ortholog prediction for disease-focused and other functional studies." BMC Bioinformatics **12**: 357.1471-2105-12-357 [pii] 10.1186/1471-2105-12-357.

Kaushik, G., A. Fuhrmann, et al. (2011). "In situ mechanical analysis of myofibrillar perturbation and aging on soft, bilayered Drosophila myocardium." Biophys J **101**(11): 2629-37.S0006-3495(11)01274-4 [pii] 10.1016/j.bpj.2011.10.042.

Kaushik, G., A. Spenlehauer, et al. (2015). "Vinculin network-mediated cytoskeletal remodeling regulates contractile function in the aging heart." Sci Transl Med **7**(292): 292ra99.7/292/292ra99 [pii] 10.1126/scitranslmed.aaa5843.

Kwon, A. T., D. J. Arenillas, et al. (2012). "oPOSSUM-3: advanced analysis of regulatory motif over-representation across genes or ChIP-Seq datasets." G3 (Bethesda) **2**(9): 987-1002.10.1534/g3.112.003202 GGG_003202 [pii].

Li, Q., X. Qi, et al. (2016). "3,3',5-triiodothyroxine inhibits apoptosis and oxidative stress by the PKM2/PKM1 ratio during oxygen-glucose deprivation/reperfusion AC16 and HCM-a cells: T3 inhibits apoptosis and oxidative stress by PKM2/PKM1 ratio." Biochem Biophys Res Commun.10.1016/j.bbrc.2016.05.030.

Marek, K. W., N. Ng, et al. (2000). "A genetic analysis of synaptic development: pre- and postsynaptic dCBP control transmitter release at the Drosophila NMJ." Neuron **25**(3): 537-47.

Molina, A. J., M. S. Bharadwaj, et al. (2016). "Skeletal Muscle Mitochondrial Content, Oxidative Capacity, and Mfn2 Expression Are Reduced in Older Patients With Heart Failure and Preserved Ejection Fraction and Are Related to Exercise Intolerance." JACC Heart Fail.10.1016/j.jchf.2016.03.011.

Monnier, V., M. Iche-Torres, et al. (2012). "dJun and Vri/dNFIL3 are major regulators of cardiac aging in Drosophila." PLoS Genet **8**(11): e1003081.10.1371/journal.pgen.1003081 PGENETICS-D-12-00162 [pii].

Moriyama, H., M. Moriyama, et al. (2014). "Role of notch signaling in the maintenance of human mesenchymal stem cells under hypoxic conditions." Stem Cells Dev **23**(18): 2211-24.10.1089/scd.2013.0642.

Neely, G. G., K. Kuba, et al. (2010). "A global in vivo Drosophila RNAi screen identifies NOT3 as a conserved regulator of heart function." Cell **141**(1): 142 53.10.1016/j.cell.2010.02.023.

Noland, R. C., T. R. Koves, et al. (2009). "Carnitine insufficiency caused by aging and overnutrition compromises mitochondrial performance and metabolic control." J Biol Chem **284**(34): 22840-52.10.1074/jbc.M109.032888.

Quinlan, C. L., I. V. Perevoshchikova, et al. (2013). "Sites of reactive oxygen species generation by mitochondria oxidizing different substrates." Redox Biol **1**: 304-12.10.1016/j.redox.2013.04.005.

Ronn, T., P. Poulsen, et al. (2008). "Age influences DNA methylation and gene expression of COX7A1 in human skeletal muscle." Diabetologia **51**(7): 1159-68.10.1007/s00125-008-1018-8.

Rothermel, B. A. and J. A. Hill (2008). "Autophagy in load-induced heart disease." Circ Res **103**(12): 1363-9.103/12/1363 [pii] 10.1161/CIRCRESAHA.108.186551.

Singh, S. H., P. Kumar, et al. (2014). "Roles of the troponin isoforms during indirect flight muscle development in Drosophila." J Genet **93**(2): 379-88.

Slaidina, M., R. Delanoue, et al. (2009). "A Drosophila insulin-like peptide promotes growth during nonfeeding states." Dev Cell **17**(6): 874-84.S1534-5807(09)00431-6 [pii] 10.1016/j.devcel.2009.10.009.

Taghli-Lamallem, O., K. Jagla, et al. (2014). "Mechanical and non-mechanical functions of Dystrophin can prevent cardiac abnormalities in Drosophila." Exp Gerontol **49**: 26-34.10.1016/j.exger.2013.10.015.

Tarasov, K. V., S. Sanna, et al. (2009). "COL4A1 is associated with arterial stiffness by genome-wide association scan." Circ Cardiovasc Genet **2**(2): 151-8.10.1161/CIRCGENETICS.108.823245.

Taylor, A. M., N. C. Berchtold, et al. (2009). "Axonal mRNA in uninjured and regenerating cortical mammalian axons." J Neurosci **29**(15): 4697-707.29/15/4697 [pii] 10.1523/JNEUROSCI.6130-08.2009.

Toba, H., L. E. de Castro Bras, et al. (2016). "Increased ADAMTS1 mediates SPARC-dependent collagen deposition in the aging myocardium." Am J Physiol Endocrinol Metab: ajpendo 00040 2016.10.1152/ajpendo.00040.2016.

Viswanathan, M. C., A. C. Blice-Baum, et al. (2016). "Cardiac-Restricted Expression of VCP/TER94 RNAi or Disease Alleles Perturbs Drosophila Heart Structure and Impairs Function." J Cardiovasc Dev Dis **3**(2).10.3390/jcdd3020019.

Viswanathan, M. C., A. C. Blice-Baum, et al. (2015). "Pseudo-acetylation of K326 and K328 of actin disrupts Drosophila melanogaster indirect flight muscle structure and performance." Front Physiol **6**: 116.10.3389/fphys.2015.00116.

Viswanathan, M. C., G. Kaushik, et al. (2014). "A Drosophila melanogaster model of diastolic dysfunction and cardiomyopathy based on impaired troponin-T function." Circ Res **114**(2): e6-17.10.1161/CIRCRESAHA.114.302028 CIRCRESAHA.114.302028 [pii].

Vogler, G. and K. Ocorr (2009). "Visualizing the beating heart in Drosophila." J Vis Exp(31).1425 [pii] 10.3791/1425.

Vojta, P. J., P. A. Futreal, et al. (1996). "Evidence for two senescence loci on human chromosome 1." Genes Chromosomes Cancer **16**(1): 55-63.10.1002/(SICI)1098-2264(199605)16:1<55::AID-GCC8>3.0.CO;2-2.

Wagner, M., B. Hampel, et al. (2001). "Metabolic stabilization of p27 in senescent fibroblasts correlates with reduced expression of the F-box protein Skp2." Exp Gerontol **37**(1): 41-55.

Wang, C. and X. Wang (2015). "The interplay between autophagy and the ubiquitin-proteasome system in cardiac proteotoxicity." Biochim Biophys Acta **1852**(2): 188-94.S0925-4439(14)00249-X [pii] 10.1016/j.bbadis.2014.07.028.

Wessells, R. J., E. Fitzgerald, et al. (2004). "Insulin regulation of heart function in aging fruit flies." Nat Genet **36**(12): 1275-81.

Wettenhall, J. M. and G. K. Smyth (2004). "limmaGUI: a graphical user interface for linear modeling of microarray data." Bioinformatics **20**(18): 3705-6.10.1093/bioinformatics/bth449 bth449 [pii].

Xu, L., D. Zheng, et al. (2014). "GCK gene-body hypomethylation is associated with the risk of coronary heart disease." Biomed Res Int **2014**: 151723.10.1155/2014/151723.

Yin, Z., X. L. Xu, et al. (1997). "Regulation of the twist target gene tinman by modular cis-regulatory elements during early mesoderm development." Development **124**(24): 4971-82.

Zambon, A. C., S. Gaj, et al. (2012). "GO-Elite: a flexible solution for pathway and ontology over-representation." Bioinformatics **28**(16): 2209-10.bts366 [pii] 10.1093/bioinformatics/bts366.
